# Bacteria export alarmone synthetases that produce (p)ppApp and (p)ppGpp

**DOI:** 10.1128/mbio.02227-25

**Published:** 2025-11-12

**Authors:** Shehryar Ahmad, Andrea G. Guedez, Berti Manisa, Abdulmalik Adewale, Kara K. Tsang, Vanessa Schiefer, Nathan P. Bullen, Harmy Thakar, Youngchang Kim, Boyuan Wang, John C. Whitney

**Affiliations:** 1 Temerty Faculty of Medicine, University of Toronto 7938, Toronto, Ontario, Canada; 2 Michael DeGroote Institute for Infectious Disease Research, McMaster University 3710, Hamilton, Ontario, Canada; 3 Department of Biochemistry and Biomedical Sciences, McMaster University 3710, Hamilton, Ontario, Canada; 4 Department of Pharmacology, UT Southwestern Medical Center 12334 , Dallas, Texas, USA; 5 Department of Infection Biology, London School of Hygiene and Tropical Medicine 4906, London, England, United Kingdom; 6 Structural Biology Center, X-ray Science Division, Advanced Photon Source, Argonne National Laboratory, Lemont, Illinois, USA; 7 David Braley Centre for Antibiotic Discovery, McMaster University, Hamilton, Ontario, Canada; Friedrich-Schiller-Universitat, Jena, Germany

**Keywords:** alarmones, bacterial protein export, (p)ppGpp, (p)ppApp, enzyme mechanism

## Abstract

Guanosine penta- and tetraphosphate [(p)ppGpp] and their adenosine analogs [(p)ppApp] are bacterial second messengers known as alarmones. Members of the RelA-SpoT homolog (RSH) family synthesize (p)ppGpp to mediate the stringent response during nutrient starvation, whereas (p)ppApp synthetases have been identified as bactericidal toxins in diverse contexts including type VI secretion systems, toxin-antitoxin modules, and phages. Although alarmone synthesis has traditionally been viewed as a cytoplasmic process, early studies in *Streptomyces* suggested the existence of secreted alarmone synthetases. Here, we identify *Sa*EAS, an exported alarmone synthetase (EAS) from *Streptomyces albidoflavus*, as the long-mysterious source of extracellular alarmone synthetase activity in *Streptomyces. Sa*EAS produces both (p)ppGpp and (p)ppApp at rates exceeding 100,000 molecules per minute and has kinetic properties adapted to low substrate environments. A broader bioinformatic survey reveals ~600 EASs linked to a range of specialized bacterial secretion systems. Characterization of two additional EASs, *Vp*EAS from *Vibrio parahaemolyticus* and *Aa*EAS from *Amycolatopsis azurea*, shows that both produce (p)ppGpp exclusively and inhibit bacterial growth when localized to the cytoplasm. These findings challenge the longstanding view of (p)ppGpp as strictly pro-survival and unveil a diverse family of secreted RSH enzymes with potential roles in interbacterial antagonism and environmental signaling.

**IMPORTANCE:**

Alarmone synthetases are intracellular enzymes that promote bacterial survival by responding to environmental stress. Although extracellular alarmone production has been reported in *Streptomyces*, the enzymes responsible for this activity remain unknown. Here, we identify hundreds of predicted exported alarmone synthetases (EASs) associated with bacterial protein export pathways. We show that *Sa*EAS, secreted by *Streptomyces albidoflavus*, efficiently synthesizes (p)ppGpp and (p)ppApp in low substrate extracellular environments. When localized to the cytoplasm, *Sa*EAS and two (p)ppGpp-specific EASs from *Vibrio parahaemolyticus* and *Amycolatopsis azurea* rapidly inhibit cell growth. Overall, our findings show that (p)ppGpp is not always a bacteriostatic, pro-survival molecule and suggest that the physiological consequences of alarmone production depend more on context and enzyme kinetics than on alarmone identity.

## INTRODUCTION

**Fig 1:**
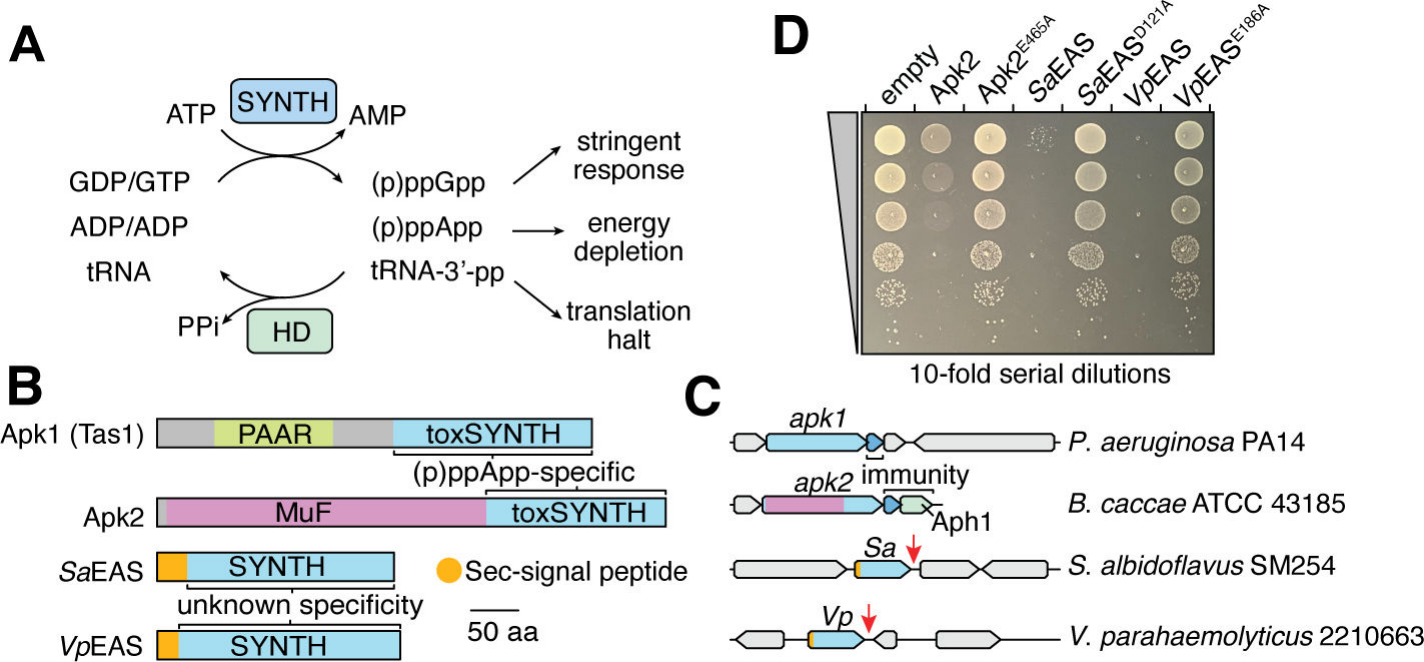
Putative Sec signal peptide-containing alarmone synthetases inhibit bacterial growth. (**A**) Schematic showing pyrophosphokinase activity and cellular effects of RSH synthetases and hydrolases in bacteria. (**B and C**) Domain architecture (**B**) and genomic context of coding loci (**C**) of two (p)ppApp-synthesizing toxSAS enzymes (Apk1 and Apk2) and signal peptide-containing *Sa*EAS and *Vp*EAS. Immunity-protein genes are highlighted for *apk1* and *apk2* in (**C**), whereas *Sa*EAS and *Vp*EAS are not followed by such genes (red arrows). (**D**) Overnight growth of *E. coli* harboring expression plasmids of the indicated alarmone synthetases. Serial dilutions of each culture were spotted on inducer-containing solid medium. Variants harboring known (Apk2) or predicted (*Sa*EAS and *Vp*EAS) active-site mutations are indicated.

Proteins belonging to the RelA-SpoT homolog (RSH) family of enzymes synthesize the nucleotides guanosine penta- and tetraphosphate (pppGpp or ppGpp, referred to as (p)ppGpp) in response to environmental stress (1). These nucleotides are 3′-pyrophosphorylation products of GTP and GDP, respectively (Fig. 1A). RSH enzymes exist either as large, multidomain proteins (so-called “long” RSH) containing both synthetase (SYNTH) and hydrolase (HD) domains (e.g., *Escherichia coli* RelA and SpoT) or as single-domain proteins known as small alarmone synthetases (SASs) and small alarmone hydrolases (SAHs) (2). (p)ppGpp triggers the stringent response in both Gram-positive and Gram-negative bacteria by inhibiting transcription, translation, and certain metabolic pathways, thereby inhibiting growth (1, 3). The hydrolase function of long RSH proteins ensures that (p)ppGpp accumulation is reversible and thus bacteriostatic (4). In *E. coli*, inducing (p)ppGpp production by either nutrient starvation or by synthetase overexpression does not affect viability (5, 6).

More recently, toxic SAS enzymes (termed “toxSAS”) have been shown to pyrophosphorylate alternative substrates, giving rise to products not recognized by canonical RSH hydrolases. For example, Apk1 (*Pseudomonas aeruginosa*), FaRel (*Cellulomonas marina*), and Apk2 (*Bacteroides caccae* and *Streptococcus pneumoniae*) synthesize (p)ppApp from ADP/ATP (4, 6–8). The highly efficient activity of these enzymes depletes ADP/ATP and consequently disrupts essential metabolic pathways that depend on these nucleotides. Other SAS enzymes, including PhRel2 (*Bacillus subtilis* la1a), FaRel2 (*Thomasclavelia ramose*, formerly *Coprobacillus* spp. D7), PhRel (*Mycobacterium* bacteriophage Phrann), and CapRel (*Mycobacterium tuberculosis* AB308), pyrophosphorylate the 3′-OH of tRNA, blocking aminoacylation and inhibiting protein synthesis (9, 10). ToxSASs are encoded alongside cognate immunity or antitoxin proteins that inhibit alarmone synthetase activity via direct protein-protein interaction or through enzymatic hydrolysis of the pyrophosphorylated nucleotide products (4, 6, 7, 9, 11). In some cases, the regulation and biological mechanism of action for toxSAS proteins have been defined. For example, Apk1 is released from its immunity protein and delivered into neighboring cells via the type VI secretion system to mediate interbacterial antagonism, whereas CapRel^SJ46^ is activated by phage proteins to limit viral replication (6, 11).

Despite this growing understanding of RSH enzyme diversity, all members characterized to date function exclusively within the bacterial cytoplasm, where ATP and acceptor nucleotides are readily available. However, several early studies of bacterial alarmones suggest that extracellular RSH enzymes also exist. For example, enzymatic activities producing (p)ppApp and (p)ppGpp were detected in the spent culture medium of *Streptomyces morookaensis*, *Actinomyces violascens*, and *Streptoverticillium septatum* (12, 13). Moreover, exogenous addition of (p)ppApp was shown to inhibit spore germination in *Streptomyces* spp., suggesting a potential signaling role for extracellular alarmones. However, the enzymes responsible for this extracellular alarmone synthetase activity and how they are secreted remain unknown (14).

In this study, we discover and characterize a subset of RSH proteins that are secreted from bacterial cells, which we term exported alarmone synthetases (EASs). Using a bioinformatic approach, we identify many EAS proteins that possess an N-terminal signal peptide required for co-translational export via the general secretory (Sec) pathway. We characterize a representative EAS from *Streptomyces albidoflavus* (*Sa*EAS) and show that it shares the SYNTH domain fold and catalytic site found in cytoplasmic SAS proteins and that it is responsible for extracellular alarmone synthesis in *S. albidoflavus*. Although *Sa*EAS synthesizes both (p)ppGpp and (p)ppApp at rates comparable to toxSAS enzymes, which are among the highest known (~100,000 molecules per minute), it lacks an associated immunity protein and does not affect *S. albidoflavus* growth. However, the expression of a signal peptide-free variant of *Sa*EAS potently inhibits bacterial growth. Similar to *Sa*EAS, an EAS from *Vibrio parahaemolyticus* (*Vp*EAS) is also non-toxic in its native state but is toxic when targeted to the bacterial cytoplasm. However, in contrast to *Sa*EAS, *Vp*EAS exclusively produces (p)ppGpp. Beyond Sec-dependent EASs, we additionally identify hundreds of other EASs linked to other protein export systems. For example, an EAS from *Amycolatopsis azurea* (*Aa*EAS) is fused to an N-terminal LXG domain that likely directs its export through the type VII secretion system (T7SS). Like *Vp*EAS, *Aa*EAS specifically synthesizes (p)ppGpp, and its expression in the cytoplasm inhibits bacterial growth. Collectively, our findings reveal the diverse substrate specificity of EAS proteins, challenge the view that (p)ppGpp is strictly bacteriostatic and pro-survival, and highlight potential new roles for EAS proteins in bacterial physiology.

## RESULTS

### Signal peptide-containing EAS enzymes synthesize alarmones and require export to prevent self-intoxication

To identify extracellular alarmone synthetases, we manually examined a previously published data set of bacterial RSH proteins for sequences containing Sec signal peptides and identified several SAS proteins in *Streptomyces* spp. and *Vibrio* spp. (Fig. 1A and B) (4). The genes encoding these proteins occur as isolated loci in their respective genomes and lack adjacent immunity protein-encoding genes, suggesting functional divergence from well-characterized toxSAS proteins such as Apk1 (Fig. 1C). Interestingly, some hits from *Vibrio* spp. had been previously annotated as “RelV,” an enzyme known to synthesize (p)ppGpp (15, 16). However, prior studies on RelV did not acknowledge the presence of a signal peptide at its N-terminus, the existence of which implies that the protein is co-translationally exported into the periplasm (15).

To investigate these enzymes further, we selected representative proteins from *Streptomyces albidoflavus* SM254 (*Sa*EAS; locus tag: Salbus254_1145; protein ID: AMM07692.1) and *Vibrio parahaemolyticus* RIMD 2210633 (*Vp*EAS; locus tag: VP1295; protein ID: HAS6678992.1) for functional analysis. When expressed in *E. coli*, signal peptide-deficient variants of *Sa*EAS (*Sa*EAS^ΔSP^) and *Vp*EAS (*Vp*EAS^ΔSP^) inhibit bacterial growth, similar to the toxSAS proteins Apk1 and Apk2 (Fig. 1D). This toxicity is dependent on enzymatic activity because active-site mutants of *Sa*EAS^ΔSP^ and *Vp*EAS^ΔSP^ fail to impair growth (Fig. 1D). We observed a similar growth inhibition phenotype with a signal peptide-deficient variant of *Vibrio cholerae* RelV (*Vc*RelV^ΔSP^; locus tag: VC1224; 70% identity to *Vp*EAS^ΔSP^) (Fig. S1A). By contrast, expression of full-length *Vp*EAS and *Vc*RelV in *E. coli* and *V. cholerae*, respectively, has no effect on growth, suggesting that the periplasmic localization of the native proteins prevents toxicity (Fig. S1A and B). Taken together, these findings demonstrate that signal peptide-containing EAS enzymes possess toxic alarmone synthetase activity, and that this toxic activity is mitigated in producing cells by co-translational EAS export through the Sec pathway.

To better understand the molecular basis for the enzymatic activity and toxicity of EASs, we next pursued structural characterization of these enzymes. However, due to their toxicity and the absence of cognate immunity proteins, we were unable to purify wild-type recombinant *Sa*EAS^ΔSP^ or *Vp*EAS^ΔSP^. Therefore, we instead expressed, purified, and crystallized a catalytically inactive double mutant of *Sa*EAS^ΔSP^ (G36-P248; L83M, D121A). The D121A substitution inactivates the enzyme by disrupting its active site, whereas the L83M mutation was introduced to facilitate selenium labeling in anticipation of using single-wavelength anomalous dispersion for overcoming the crystallographic phase problem. We successfully solved the structure of this *Sa*EAS^ΔSP^ variant (hereafter *Sa*EAS*^ΔSP^) at 2.4 Å resolution using molecular replacement with an AlphaFold2-predicted structure as the search model (17). The final structure of *Sa*EAS*^ΔSP^ was refined to an *R*_work_ and *R*_free_ of 23.8% and 27.6%, respectively (Table S1).

**Fig 2:**
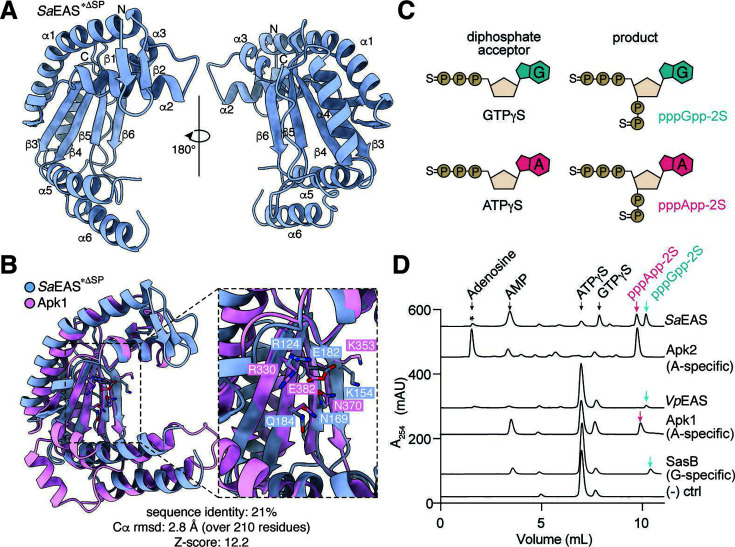
*Sa*EAS and *Vp*EAS resemble toxSAS enzymes and synthesize bacterial alarmones. (**A**) Two views of a 2.4 Å resolution *Sa*EAS*^ΔSP^ (L83M, D121A) crystal structure shown in ribbon representation. Secondary structure elements (α1–α6, β1–β6) and N- and C-termini are labeled. (**B**) Structural alignment between *Sa*EAS*^ΔSP^ (L83M, D121A) (blue) and the toxin domain of Apk1 (pink, PDB: 6OX6). (**C**) Cartoon diagram of PPi acceptors and reaction products for alarmone synthetase reactions containing GTPγS (top) and ATPγS (bottom). (**D**) Anion-exchange traces of cell lysates containing *Sa*EAS or *Vp*EAS incubated with 5 mM ATPγS and 1 mM GTPγS at 37°C for 5 min. Traces from reactions using an Apk2-containing lysate or purified recombinant Apk1 or SasB are used to highlight peak positions of pppApp-2S or pppGpp-2S. The Apk2 lysate was incubated with ATPγS and GTPγS for 60 min, during which AMP produced by Apk2 is converted into adenosine (asterisk) likely by native endogenous *E. coli* phosphatases, while pppApp-2S remains stable. Negative control reaction [indicated as (−) ctrl] does not contain enzyme.

The overall structure of *Sa*EAS*^ΔSP^ reveals a canonical SYNTH domain fold comprising a central β-sheet composed of four antiparallel β-strands that is flanked by six α-helices (Fig. 2A). Despite possessing only 21% sequence identity, the structure of *Sa*EAS*^ΔSP^ most closely resembles that of Apk1 (PDB: 6OX6), with a Z-score of 12.2 and RMSD of 2.8 Å across 210 aligned residues (Fig. 2B) (6). The active-site architecture of the two enzymes is also remarkably conserved, with nearly identical positioning of residues involved in ATP binding between *Sa*EAS and Apk1 (*Sa*EAS/Apk1: R124/R330, E182/E382, Q184/Q382, N169/N370, K154/K353) (2, 6, 18). Overall, these structural findings demonstrate that *Sa*EAS adopts a conserved SAS fold with a canonical active-site architecture, which supports its functional classification as an exported alarmone synthetase.

To determine the alarmone specificity of *Sa*EAS and *Vp*EAS, we next reconstituted their enzymatic activities *in vitro* using lysates from *E. coli* cells expressing recombinant *Sa*EAS or *Vp*EAS. Reactions were carried out using the non-hydrolyzable thiophosphates ATPγS and GTPγS because these substrates have enhanced stability in the presence of endogenous *E. coli* phosphatases but do not interfere with the pyrophosphokinase reaction (Fig. 2C). Analysis of the reaction products by anion-exchange chromatography revealed that *Vp*EAS specifically uses GTPγS as an acceptor nucleotide to produce pppGpp-2S, which is consistent with previous findings on *Vc*RelV and canonical SAS enzymes involved in the stringent response, such as *Bacillus subtilis* SasB (Fig. 2D) (19). In contrast, *Sa*EAS produced both pppApp-2S and pppGpp-2S, similar to the promiscuous extracellular alarmone synthetase activity previously described for several *Streptomyces* species (Fig. 2D) (12, 13). Together, these results show that signal peptide-containing EAS enzymes function as *bona fide* alarmone synthetases but harbor distinct substrate preferences that may reflect divergent physiological roles.

### *Sa*EAS is responsible for extracellular alarmone synthetase activity in *S. albidoflavus*

**Fig 3:**
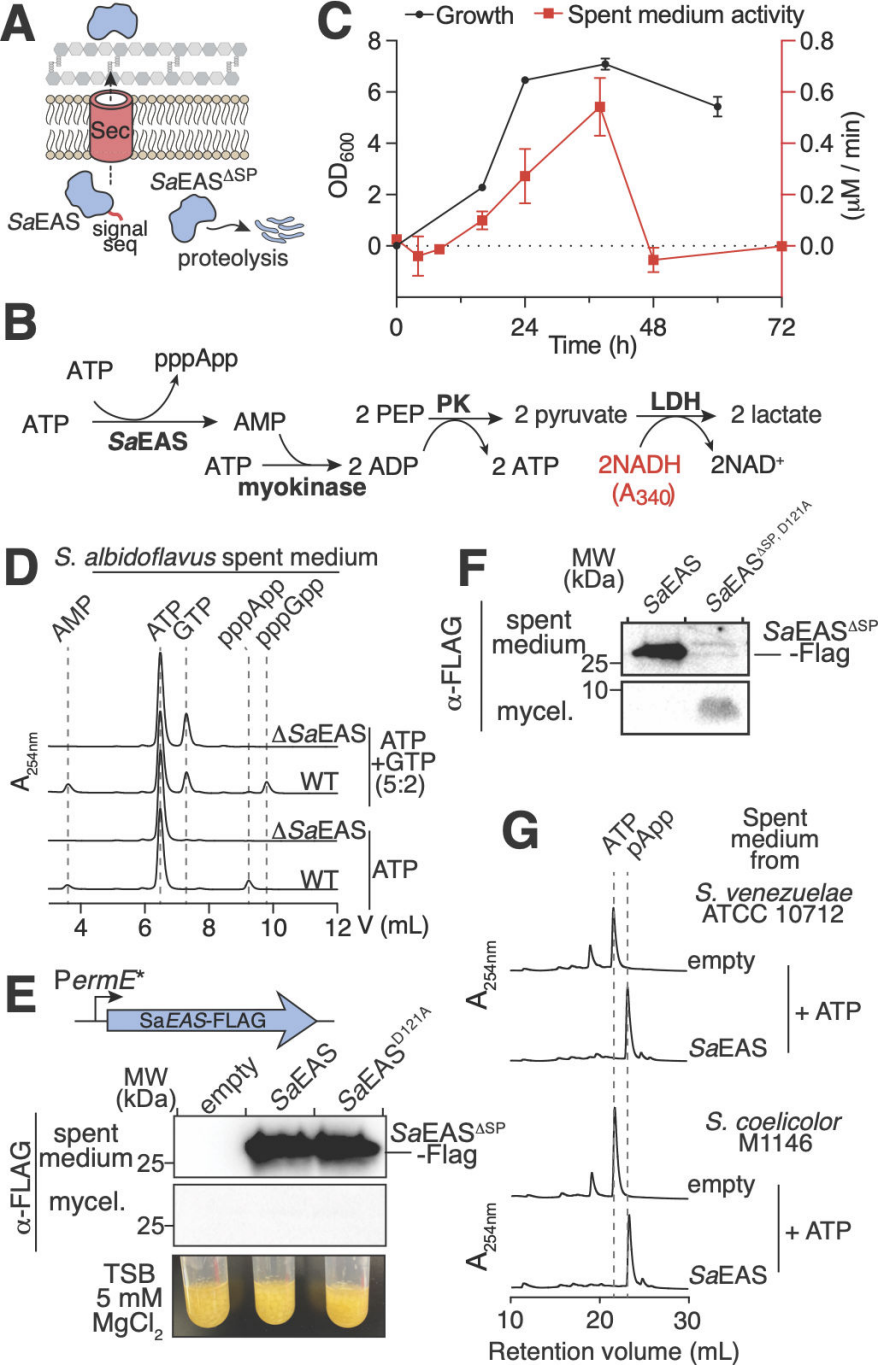
*Sa*EAS is an extracellular alarmone synthetase produced by *S. albidoflavus*. (**A**) Diagram showing co-translational secretion of the signal peptide (red)-containing *Sa*EAS and proteolytic degradation of cytoplasmic *Sa*EAS^ΔSP^. (**B**) Schematic of enzymatic coupling of AMP production to NADH consumption for real-time monitoring of alarmone synthesis using 340 nm absorbance (PK, pyruvate kinase; LDH, lactate dehydrogenase). (**C**) Cell density and pppApp-synthesis activity in spent media as a function of time for wild-type *S. albidoflavus* SM254 in TSB liquid cultures. pppApp synthesis was measured using the assay described in panel **B**. Data show mean ± SD of *n* = 3 biological replicates. (**D**) Anion-exchange traces showing nucleotides in the supernatant of either wild-type *S. albidoflavus* (WT) or a ∆*Sa*EAS mutant incubated with either 5 mM ATP + 2 mM GTP or 5 mM ATP. (**E and F**) Western blot of spent medium or mycelium (mycel.) fractions from *S. albidoflavus* (∆*Sa*EAS strain) constitutively expressing an empty vector or the indicated *Sa*EAS constructs after 36 h of growth in TSB liquid medium. The signal sequence is cleaved upon export, and a signal sequence-deficient variant (*Sa*EAS^∆SP^-FLAG) is detected in the spent medium. In panel **E**, cultures were grown for up to 120 h to assess for (p)ppApp-dependent toxicity, and no differences were observed (bottom panel). (**G**) Anion-exchange traces for 1 mM ATP treated with spent medium from the indicated *Streptomyces* species overexpressing either an empty vector or *Sa*EAS-FLAG.

The presence of a signal peptide, a hallmark of extracellular localization, suggests that orthologs of *Sa*EAS are responsible for the previously reported extracellular alarmone synthetase activity in *Streptomyces* (Fig. 3A) (12, 13). Since extracellular synthetase activity has not been reported for *S. albidoflavus*, we first cultured strain SM254 in tryptic soy broth, sampled the spent medium over a time course, and measured (p)ppApp synthetase activity using an enzyme-coupling assay that links AMP production to NADH consumption for colorimetric detection (Fig. 3B). This assay revealed that (p)ppApp synthetase activity is present in the spent medium of *S. albidoflavus*, increases with cell density, and decreases in stationary phase, similar to the reported findings for *S. morookaensis* (Fig. 3C) (13). Interestingly, the addition of ATP and GTP to the spent medium resulted primarily in pppGpp synthesis, with pppApp production occurring at a similar efficiency only in the absence of GTP (Fig. 3D). Finally, deletion of the *Sa*EAS-encoding gene from the *S. albidoflavus* chromosome using the pCRISPomyces system abolished extracellular alarmone synthetase activity, indicating that *Sa*EAS is the sole gene product responsible for alarmone production in *S. albidoflavus* supernatants (Fig. 3D) (20).

Compared to *S. morookaensis*, the (p)ppApp synthetase activity observed in *S. albidoflavus* spent medium is nearly 100-fold lower, suggesting that either the wild-type SM254 strain and/or the culture conditions disfavor *Sa*EAS expression or secretion. To investigate this further, we fused the *SaEAS* promoter (P*_SaEAS_*) to a glucuronidase-encoding gene, *gusA*, which is used as colorimetric transcriptional reporter in *Streptomyces* spp., and observed low levels of P*_SaEAS_-gusA* expression (Fig. S2A) (21). By contrast, fusion of the constitutively expressed erythromycin resistance gene (ermE*) promoter (P_ermE__*_) to *gusA* resulted in *S. albidoflavus* blue-pigmented colonies in the presence of X-gluc, indicating high levels of transcription from this promoter (Fig. S2A) (22). Therefore, to develop an overexpression system to study *Sa*EAS secretion and enzymatic function, we fused P_ermE__*_ to FLAG-tagged variants of *Sa*EAS or its active-site mutant *Sa*EAS^D121A^-FLAG (Fig. 3E). Each construct was integrated into a neutral chromosomal site in an *S. albidoflavus* strain lacking its native copy of *Sa*EAS (*S. albidoflavus* SM254 ∆*Sa*EAS). Consistent with *Sa*EAS undergoing Sec-dependent co-translational export from *S. albidoflavus* cells, FLAG tag-specific immunoblotting detects both *Sa*EAS-FLAG and *Sa*EAS^D121A^-FLAG in the spent medium but not in the mycelium (Fig. 3E). Upon secretion, both proteins appear to lack their signal peptides because they exhibit an identical migration pattern on SDS-PAGE as a *Sa*EAS^ΔSP, D121A^-FLAG variant expressed and purified from *E. coli* (Fig. S2B and C). Notably, despite being toxic in the cytoplasm of *E. coli*, extracellular *Sa*EAS-FLAG did not exhibit any toxicity toward *S. albidoflavus* over several days of growth (Fig. 3E, bottom panel). To test whether the predicted Sec signal peptide is essential for *Sa*EAS secretion, we expressed the inactive, signal peptide-free *Sa*EAS^ΔSP, D121A^-FLAG in *S. albidoflavus*. No FLAG-tag signal was detected in the spent medium; instead, a weak signal appeared in the mycelial fraction, corresponding to a lower-molecular-weight degradation product (Fig. 3F). This observation suggests that mislocalized *Sa*EAS is rapidly degraded by the cell, suggesting that there exists a proteolytic mechanism that protects cells from unintended cytoplasmic *Sa*EAS.

We next inserted the *Sa*EAS-FLAG expression construct into a neutral chromosomal site in *Streptomyces venezuelae* ATCC 10712 and *Streptomyces coelicolor* M1146, two strains that lack a *Sa*EAS ortholog, to test whether *Sa*EAS is secreted and enzymatically active when heterologously expressed. Indeed, *Sa*EAS-FLAG was efficiently secreted from *S. venezuelae* (Fig. S2D), and high-level expression from P_ermE*_ resulted in robust (p)ppApp synthetase activity in *S. venezuelae* spent medium, with ATP being completely converted to pApp within 30 min (Fig. 3G). pApp is the pyrophosphokinase product of AMP and pppApp, both of which are generated from ATP, and the use of pppApp as a PPi donor has also been reported for Apk1 and other *Streptomyces* extracellular alarmone synthetases (Fig. S2E) (6, 12). These findings confirm that *Sa*EAS secretion is mediated by the broadly conserved Sec pathway, and that alarmone synthetase activity in spent media is dictated primarily by expression levels of *Sa*EAS.

### *Sa*EAS is a promiscuous and highly efficient extracellular alarmone synthetase

**Fig 4:**
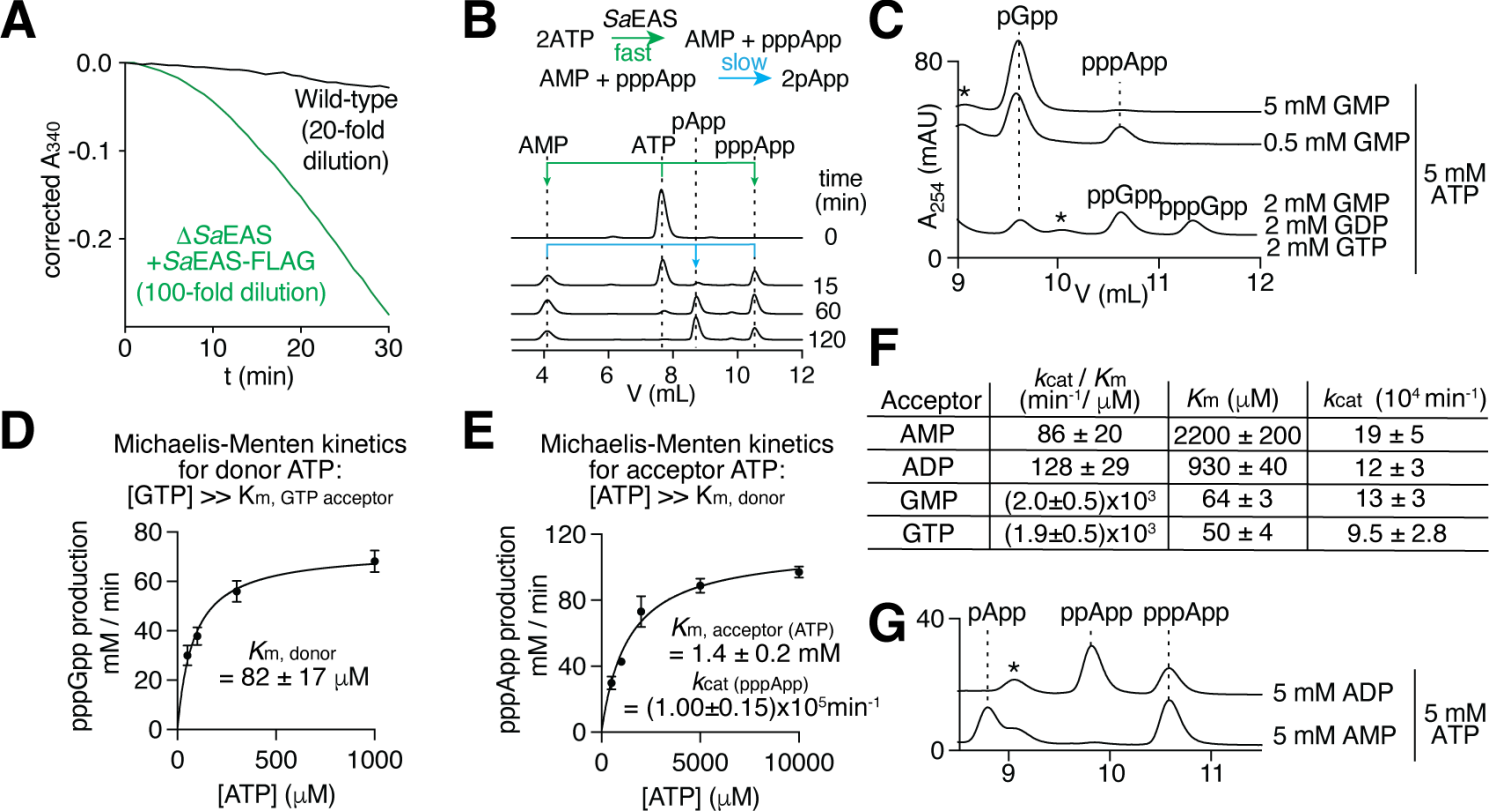
*Sa*EAS is a highly efficient alarmone synthetase. (**A**) Monitoring pppApp synthesis over time in the spent medium of either wild-type *S. albidoflavus* or the ∆*Sa*EAS strain overexpressing *Sa*EAS-FLAG (see Fig. 3B for coupled assay schematic). Background signal from ADP-producing phosphatase or kinase activity in each sample was measured using a separate reaction without myokinase (decoupling the produced AMP from NADH consumption) and subtracted. The samples were diluted by the indicated amounts to track the reactions over a 30 min period. (**B**) Anion-exchange traces of ATP treated with spent medium containing *Sa*EAS^ΔSP^-FLAG highlighting synthetase preference for ATP as a PPi donor over pppApp. (**C**) Anion-exchange traces showing alarmones produced from the indicated mixture of ATP and guanylate PPi acceptors. (**D and E**) Initial velocity of pppGpp synthesis from 5 mM GTP as the PPi acceptor (**D**) and pppApp synthesis in the absence of other PPi acceptors (**E**) as a function of ATP concentration. Data represent mean ± range from *n* = 2 technical replicates. Fitting results are shown as mean ± SD for *K*_*m*_. (**F**) *k*_cat_/*K*_*m*_, *K*_*m*_, and *k*_cat_ values for indicated PPi acceptors. GDP is omitted because ppGpp co-elutes with pppApp and thus cannot be quantified. Data represent mean ± SD: main source of error is the fitting error of *V*_max_ and *K*_*m*_ values for pppApp and pppGpp synthesis involved in calculation. See Materials and Methods for details. (**G**) Same as panel **C**, except that reactions contained ATP and other adenylate nucleotides.

Consistent with high-level expression driven by P_ermE*_, spent medium from *S. albidoflavu*s cells overexpressing *Sa*EAS-FLAG exhibits markedly higher (p)ppApp synthetase activity than wild-type cultures (Fig. 4A). Using recombinant *Sa*EAS^ΔSP, D121A^-FLAG as an internal standard for quantitative α-FLAG immunoblotting, we estimated the concentration of secreted *Sa*EAS^ΔSP^-FLAG in the spent medium to be ~15 nM (Fig. S2C). When incubated with ATP, the supernatant initially generated AMP and pppApp, which were subsequently converted to pApp (Fig. 4B). Importantly, there was no detectable increase in ADP or ppApp over time, indicating minimal phosphatase or ATPase activity in the medium (Fig. 4B). We therefore conducted kinetic analyses of *Sa*EAS directly on spent medium, without further purification. Using ATP as the pyrophosphate (PPi) donor, we found that *Sa*EAS preferentially uses guanylate nucleotides (GMP, GDP, and GTP) rather than ATP as PPi acceptors, with little selectivity among them (Fig. 4C). The *K*_*m*_ values for ATP as a PPi donor and guanylate nucleotides as PPi acceptors were all below 100 µM, which is considerably lower than those reported for cytoplasmic synthetases such as *E. coli* RelA and *B. subtilis* SasB (Fig. 4C through F) (23, 24). This may reflect an adaptation to the lower substrate concentrations typical of the extracellular environment. By contrast, *K*_*m*_ values for adenosine-based acceptors (AMP, ADP, ATP) were 20–30 times higher than those for guanylate acceptors (Fig. 4F and G). *Sa*EAS could not use GTP, CTP, or UTP as PPi donors, nor could it use CTP, UTP, or dGTP as acceptors, which was determined based on the absence of product formation prior to ATP depletion for pppApp synthesis. This substrate specificity profile resembles the previously reported extracellular synthetase activity in other *Streptomyces* species (12, 13). Strikingly, quantification of enzyme activity revealed catalytic rates (*k*_cat_) of 190,000 min^−1^ (pApp), 120,000 min^−1^ (ppApp), 100,000 min⁻¹ (pppApp), 130,000 min^−1^ (pGpp), and 95,000 min⁻¹ (pppGpp) at pH 7.5, ranking *Sa*EAS as the most efficient bifunctional alarmone synthetase identified to date, with a catalytic rate comparable to the (p)ppApp-specific synthetase Apk1 (6). Overall, these findings establish *Sa*EAS as a potent and promiscuous extracellular alarmone synthetase that is well-suited for functioning in substrate-limited environments.

### Cytoplasmic alarmone overproduction by EAS proteins is highly toxic

The Sec pathway directs *Sa*EAS, and likely *Vp*EAS, to cellular compartments with low nucleotide availability—the extracellular space and periplasm, respectively. This localization raises the question of how these enzymes, which require nucleotide substrates for their activities, function in such environments. Although the existence of extracellular 5′-phosphorylated nucleotides is theoretically possible, particularly following stochastic events like cell lysis, it is unclear why an enzyme would evolve to sense such unpredictable events. Indeed, all reported extracellular alarmone synthetase activities in *Streptomyces* have required the exogenous addition of nucleotide substrates to measure (12, 13). An alternative explanation is that exported EAS proteins enter the cytoplasm of neighboring cells to exert their effects, akin to secreted antibacterial toxins such as colicins, which translocate across bacterial membranes via receptor-mediated uptake (25). Such diffusible antibacterial toxins typically are species-specific due to their reliance on surface receptors. As described above, expression of recombinant *Sa*EAS in *S. venezuelae* ATCC 10712 and *S. coelicolor* M1146 resulted in successful enzyme export and did not impact growth of either species, suggesting that these strains are not targets of the synthetase. Some *Streptomyces* species have been shown to antagonize *Bacillus* during co-culture, raising the possibility that *Sa*EAS or its orthologs participate in interbacterial antagonism (26). To test this, we co-cultured *Bacillus subtilis* with *S. albidoflavus* strains overexpressing *Sa*EAS-FLAG on solid media. However, the overproduction of extracellular *Sa*EAS-FLAG had no measurable effect on *B. subtilis* growth under our experimental conditions, suggesting a lack of uptake or susceptibility (Fig. S3).

**Fig 5:**
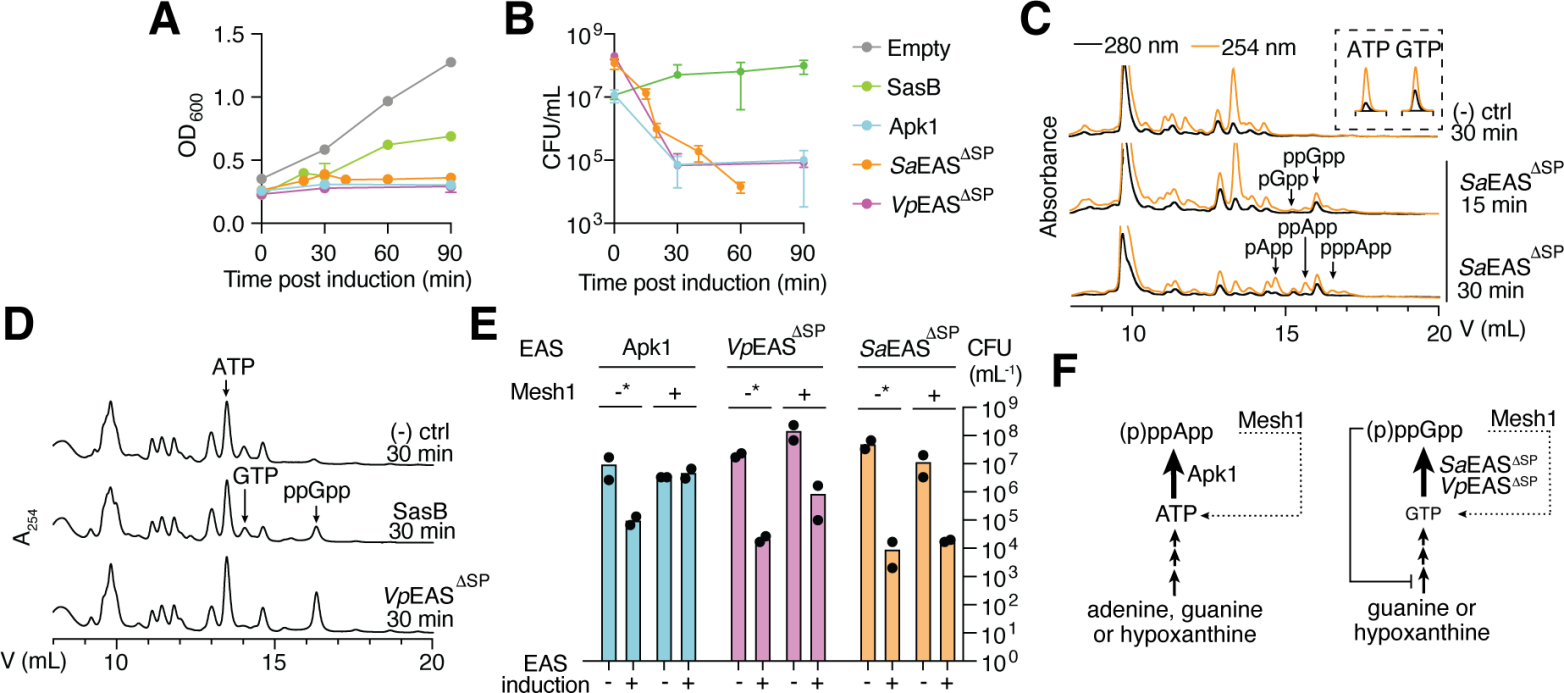
Cytoplasmic expression of *Sa*EAS and *Vp*EAS is lethal due to alarmone overproduction. (**A and B**) Time course of *E. coli* culture growth (optical density) (**A**) and CFUs (**B**) after induction of the indicated alarmone synthetases during exponential-phase growth. Data are mean ± SD of *n* = 3 biological replicates. (**C and D**) Anion-exchange traces of metabolite extracts from *E. coli* after expression of the indicated alarmone synthetases. (-) ctrl indicates cells harboring an empty expression vector treated under the same conditions. The inset in panel **C** highlights the distinct absorbance profiles between adenosine and guanosine nucleotides using the absorbance ratio between 254 and 280 nm wavelengths for ATP and GTP. (**E**) CFU quantification of *E. coli* constitutively expressing either Mesh1 or catalytically inactive Mesh1^E65A^ (indicated as −*). Samples were collected 30 min post-induction of the indicated alarmone synthetases. Data show the mean of *n* = 2 biological replicates. (**F**) The inhibition of GTP synthesis by (p)ppGpp exacerbates GTP depletion through uncontrolled (p)ppGpp synthetase activity.

To directly examine the consequences of cytoplasmic accumulation, we expressed *Sa*EAS^ΔSP^ and *Vp*EAS^ΔSP^ in *E. coli*. Remarkably, 30 min of expression of either alarmone synthetase caused a severe reduction in colony-forming units (CFUs) (Fig. 5A and B). This contrasts with previously characterized bacteriostatic alarmone synthetases such as SasB, which inhibit growth without affecting cell viability in *E. coli*, and aligns more closely with the potent toxicity of the secreted T6SS effector, Apk1 (6, 7). To understand the underlying basis for cell death, we performed anion-exchange chromatography on metabolites extracted from *E. coli* following EAS^ΔSP^ induction over time. *Sa*EAS^ΔSP^ expression led to a rapid accumulation of ppGpp in the first 15 min, followed by a marked increase in (p)ppApp that likely occurred due to guanylate substrate depletion (Fig. 5C). By contrast, *Vp*EAS^ΔSP^ expression resulted in exclusive accumulation of ppGpp, consistent with its guanylate-specific substrate preference (Fig. 5D).

To our knowledge, *Vp*EAS^ΔSP^ is the first (p)ppGpp synthetase that inhibits *E. coli* viability, contradicting the generally accepted bacteriostatic effects of (p)ppGpp (1). To investigate this further, we co-expressed human Mesh1, an alarmone hydrolase capable of degrading both (p)ppGpp and (p)ppApp (27, 28). Mesh1 expression reduced the bactericidal effects of Apk1 expression by two orders of magnitude, which is consistent with previous findings made for the (p)ppApp-specific hydrolase *Pa*SAH (Fig. 5E) (29). However, Mesh1 was less effective at restoring viability to *Sa*EAS^ΔSP^- and *Vp*EAS^ΔSP^-expressing cells (Fig. 5E), suggesting a distinct toxicity mechanism. We hypothesize that ppGpp produced by *Sa*EAS^ΔSP^ and *Vp*EAS^ΔSP^ exerts toxicity through both direct GTP depletion and inhibition of GTP synthesis (Fig. 5F). Unlike (p)ppApp, which does not inhibit ATP synthesis through purine salvage, ppGpp inhibits key biosynthetic pathways that feed into GTP production in *E. coli* (30). Inhibition of such pathways, combined with the GTP consumption required for (p)ppGpp synthesis, may irreversibly reduce GTP levels below the threshold required for viability, even in the presence of Mesh1. Overall, our results show that when localized to the cytoplasm, *Sa*EAS and *Vp*EAS render *E. coli* non-culturable due to alarmone overproduction and metabolite depletion.

### EAS proteins are associated with diverse protein export pathways in bacteria

To investigate the conservation of EAS proteins in *Streptomyces*, we searched for *Sa*EAS homologs across this bacterial genus using phmmer and performed gene synteny analysis on the resulting hits (31). A neighbor-joining tree of 152 hits grouped these SYNTH domain-containing proteins (≥35% sequence identity) into five major clades (Fig. S4A; see File S1 for sequences). Genes in clusters C, D, and E are scattered across different chromosomal loci and do not occupy a conserved position within *Streptomyces* genomes (Fig. S4B and C). Like *Sa*EAS, these genes are not predicted to be co-operonic with small open reading frames encoding putative immunity proteins. SignalP predicts a Sec-type signal peptide in many sequences in cluster C (19/32) and in nearly all members of cluster D (29/31) and cluster E (60/61, including *Sa*EAS) (32). By contrast, proteins in clusters A and B (28 total sequences) lack a signal peptide and are encoded within conserved gene clusters (Fig. S4B and C). For example, a cluster A homolog from *Streptomyces albireticuli* co-occurs with genes encoding the conjugation-associated proteins TrwB, TrwK, and TrwI, suggesting involvement with a type IV secretion system (33, 34). Similarly, cluster B homologs are consistently found adjacent to genes encoding putative immunity proteins and structural components of type VII secretion systems, an arrangement reminiscent of toxSAS loci such as type VI secretion system-associated *apk1*, and consistent with the established role of T7SS in mediating interbacterial antagonism in Gram-positive bacteria (6, 35–37). Collectively, these findings suggest that *Streptomyces* species release alarmone synthetases through multiple secretion systems, and therefore, the export of these extracellular enzymes is not limited to the Sec pathway. We refer to all such proteins, regardless of pathway, as exported alarmone synthetases.

**Fig 6:**
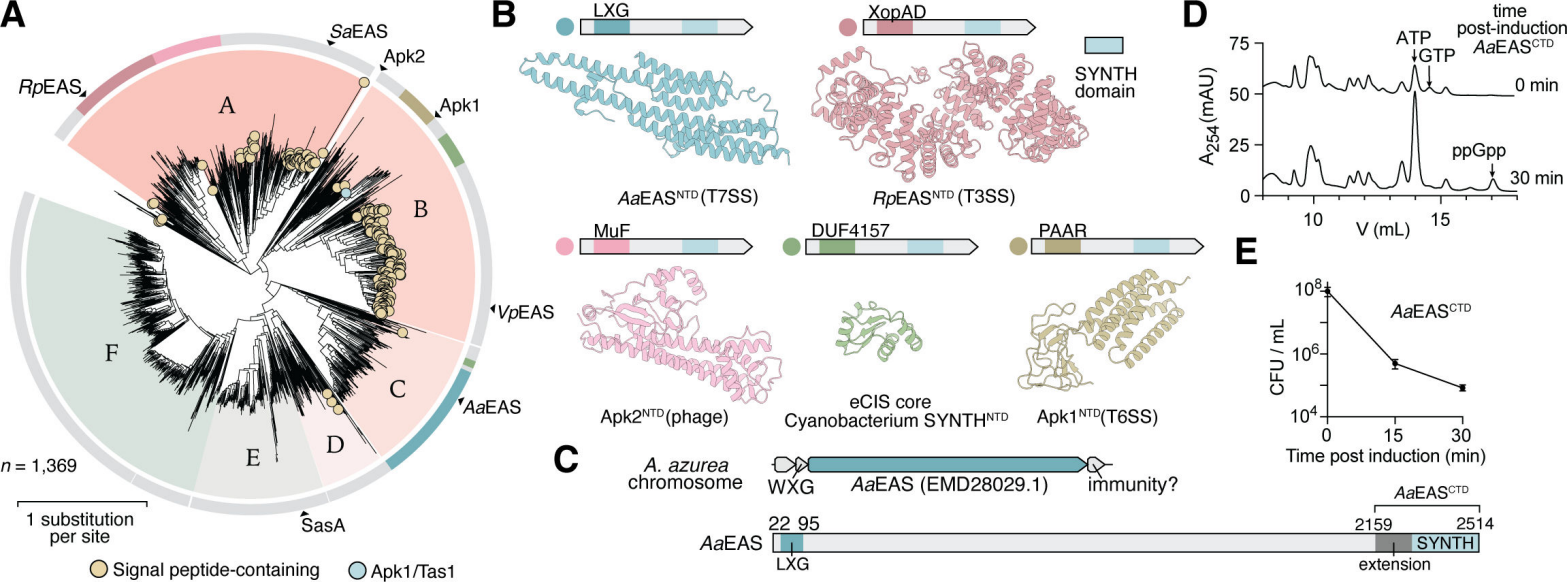
Identification of EAS enzymes associated with diverse protein secretion systems in bacteria. (**A**) Distribution of 1,369 non-redundant Apk1 homologous sequences in bacteria. A total of six clusters (A–F; ≥20 or more sequences with ≥ 24.5% sequence identity) were identified and are denoted using different colors. Tan circles represent sequences with predicted Sec signal peptides (252 sequences), and the blue circle represents Apk1/Tas1. If the exact sequence was excluded from the clustering analysis (Apk2, *Sa*EAS, *Vp*EAS, and SasA), a representative sequence with high homology was instead indicated. The outer ring highlights the subclusters containing unique amino-terminal recognition domains for specialized secretion systems. The scale bar indicates one amino acid substitution per site. (**B**) Domain architecture of selected EAS sequences and AlphaFold3 structural prediction of the N-terminal secretion system recognition domain. Each domain is colored according to the corresponding subclusters identified in panel **A**. The MuF cluster consists primarily of sequences found in *Streptococcus* species. Apk2 is used as a representative sequence to show the MuF N-terminal recognition domain structure and is not part of a subcluster due to a limited number of similar sequences (<20). (**C**) Genomic region and linear protein diagram for a type VII secretion system-associated EAS protein, *Aa*EAS from *Amycolatopsis azurea* (protein ID WP_005154424.1). The *Aa*EAS^CTD^ indicates the construct used for downstream analysis (see Fig. S5A for Alphafold3 model). (**D**) Anion-exchange traces of hydrophilic metabolite extract from *E. coli* pre- and post-induction of *Aa*EAS^CTD^. (**E**) CFU enumeration of *E. coli* culture as a function of time post-induction of *Aa*EAS^CTD^. Data are mean ± SD for *n* = 3 biological replicates.

To broaden this analysis beyond *Streptomyces* species, we performed an iterative profile HMM sequence similarity search using jackhmmer with Apk1 as the query sequence (7). The search was stopped after two iterations to avoid the inclusion of large, multidomain RSH proteins that emerged in subsequent iterations (File S2). After removing sequences with ≥95% identity and filtering for unique SYNTH-domain-containing sequences, a final set of 1,369 non-redundant EAS candidates was used to construct a maximum-likelihood phylogenetic tree (Fig. 6A; Files S3 and S4). Sequences with ≥25% identity were grouped into six major clusters (labeled A–F). Signal sequence prediction identified 252 of these sequences as encoding a Sec signal peptide, primarily in cluster A (*Streptomyces*, including *Sa*EAS) and cluster B (*Vibrio* and *Shewanella*, including *Vp*EAS) (Fig. 6A; tan circles). Notably, no twin-arginine translocation (Tat) signal sequences were detected in any clusters, likely reflecting an incompatibility between the Tat pathway, which requires protein folding prior to export, and the presumed toxicity of folded SYNTH domains in the cytoplasm.

In contrast to the α-helical transmembrane signal sequences used by Sec and Tat pathways, many specialized bacterial secretion systems rely on the recognition of folded three-dimensional protein domains to recruit their substrates for export (38). To investigate whether EAS proteins might exploit such export mechanisms, we analyzed our full sequence data set using remote homology detection and structural prediction tools to identify conserved domains associated with bacterial secretion systems (39–41). In addition to Sec signal peptides, we identified five distinct export-associated domains previously linked to diverse bacterial secretion systems (Fig. 6B). The WXG/LXG domain was the most prevalent, found in 85 sequences (6.2%). This motif is characteristic of effectors exported by type VII secretion systems (T7SS), which are known to mediate interbacterial antagonism in Gram-positive bacteria (36, 42, 43). The XopAD/9G6K domain, a recognition module composed of SKWP repeats found in *Xanthomonas* type III secretion system (T3SS) effectors, is detected in 69 sequences (5%) (44). Notably, previous studies have identified Rel-like domains in some XopAD-containing proteins, and our findings suggest that EAS proteins contribute to this growing family of putative T3SS-exported alarmone synthetase effectors (44, 45). The SPP1/MuF domain, present in 39 sequences (2.8%), is a minor phage capsid component and is found in phage-encoded SAS proteins such as Apk2 (7, 8). The DUF4157 domain, observed in seven sequences (0.05%), is a hallmark of effectors secreted via extracellular contractile injection systems, which deliver toxic payloads into contacting cells (46, 47). Finally, the Rhs/PAAR domain was identified in 15 sequences (1%) and is associated with substrates of type VI secretion systems, including the effector protein Apk1 (6). Overall, these results reveal that EAS proteins are functionally linked to a broad array of bacterial export pathways. The widespread presence of secretion system-associated domains within the EAS family of proteins suggests a modular strategy for intercellular delivery in diverse ecological contexts.

### A T7SS-associated EAS synthesizes (p)ppGpp and inhibits bacterial growth

To further explore the functional diversity of exported alarmone synthetases, we focused on *Aa*EAS, a representative EAS protein from *Amycolatopsis azurea* that is associated with a T7SS and contains an N-terminal LXG domain characteristic of T7SS substrates (Fig. 6B) (36). Structural prediction by AlphaFold3 indicates that the C-terminal SYNTH domain of *Aa*EAS likely folds together with approximately 120 upstream residues to form an extended structural unit (Fig. 6C; Fig. S5A). We therefore expressed this extended SYNTH domain (*Aa*EAS^CTD^) in *E. coli* for functional characterization.

Expression of *Aa*EAS^CTD^ in *E. coli* resulted in substantially decreased CFUs and a concomitant increase in intracellular ppGpp levels (Fig. 6D through E and Fig. S5B). Using lysate of these intoxicated cells and ATPγS and GTPγS as substrates, we observed (p)ppGpp-specific synthetase activity using anion-exchange chromatography (Fig. S5C). These findings closely resemble those observed for *Vp*EAS^ΔSP^, suggesting that *Aa*EAS^CTD^ employs a similar intoxication mechanism through overproduction of guanosine-containing alarmones. *Aa*EAS is encoded downstream of a gene predicted to encode a T7SS targeting factor, known to recruit proteins to the T7SS, and is encoded upstream of a small open reading frame that likely encodes a cognate immunity protein commonly found with T7SS antibacterial effectors (Fig. 6C) (48). This genetic linkage supports the hypothesis that *Aa*EAS functions as an antibacterial effector in its native context, where it likely intoxicates neighboring cells by inducing (p)ppGpp accumulation. In sum, *Aa*EAS represents a second, mechanistically validated example of an exported alarmone synthetase capable of reducing bacterial viability through (p)ppGpp overproduction, reinforcing the role of EAS proteins as versatile effectors deployed by diverse bacterial secretion systems.

## DISCUSSION

In this study, we identified *Sa*EAS as the enzyme responsible for the long-reported but previously uncharacterized extracellular alarmone synthetase activity in *Streptomyces* species. Kinetic analysis revealed that *Sa*EAS has a remarkably low *K*_*m*_ for ATP as the pyrophosphate donor and a high *k*_cat_ for both (p)ppGpp and (p)ppApp synthesis, consistent with its adaptation to function in extracellular environments that contain low levels of nucleotide substrate. Additionally, we showed that cytoplasmic expression of *Vp*EAS and *Aa*EAS induces ppGpp accumulation without detectable (p)ppApp production and yet still renders cells non-culturable. These findings challenge the prevailing view that (p)ppGpp is exclusively a bacteriostatic and pro-survival molecule.

### Adaptation of *Sa*EAS to extracellular environments

*Sa*EAS and other signal peptide-containing EAS enzymes are targeted for secretion from the cytoplasm into compartments where nucleotide substrates are presumed to be scarce. Because our data demonstrate that *Sa*EAS and *Vp*EAS are functional alarmone synthetases, we presume they must either act in the extracellular milieu using non-canonical sources of substrates or enter the cytoplasm of nearby cells where nucleotide substrates are abundant. For *Sa*EAS, its low *K_m_* values for ATP (80 µM) and GDP (25 µM) support the former scenario because they are well below those of typical cytoplasmic synthetases and likely reflect adaptation to the low nucleotide concentrations found outside cells (23, 24). Additionally, a *k*_cat_ approaching 10^5^ min^−1^ places *Sa*EAS among the most active RSH enzymes characterized to date and ensures that any low-abundance substrates encountered by the enzyme are rapidly converted to their corresponding alarmone products. We also observed that *S. albidoflavus* spent medium lacks detectable phosphatase activity, suggesting a stable extracellular environment conducive to alarmone accumulation. Where might these extracellular nucleotide substrates come from? Mycelial lysis is a well-documented phenomenon among *Streptomyces* species, which often colonize microenvironments that restrict solute diffusion, such as plant root tissues (49, 50). Lysis in such environments may release nucleotides at considerable concentrations. Additionally, many *Streptomyces* produce antibiotics capable of lysing competitor microbes, providing another potential substrate source (51). Thus, extracellular alarmones may function as indicators of nearby cell lysis events that result from antibiotic-mediated killing or interbacterial antagonism mediated by antibacterial proteins, such as the recently described *Streptomyces* umbrella toxins (52). We envision that *Streptomyces* and neighboring organisms may sense extracellular alarmones using cell-surface receptors or by importing these nucleotides to trigger the cytoplasmic stringent response.

The ability of *Sa*EAS to rapidly synthesize (p)ppGpp under low substrate concentrations, however, does not rule out the possibility of cytoplasmic re-entry of the enzyme. For instance, *S. albidoflavus* may use *Sa*EAS to target closely related *Streptomyces* for resource competition. This would parallel the role of Apk1 from *Pseudomonas aeruginosa* PA14, a (p)ppApp-producing, T6SS-dependent EAS that enters and antagonizes *P. aeruginosa* PAO1 cells (6). Such a mechanism would require the host organism, *S. albidoflavus*, to carry a cytoplasmic immunity protein against *Sa*EAS, which we did not identify using structure- and sequence-based analyses of its neighboring genes. Experiments to test this directly were hindered by our observation that the active, signal peptide-deficient variant of *Sa*EAS was too toxic to transform into *E. coli* ET12576, the strain typically used for conjugation with *Streptomyces*. Therefore, it remains possible that *Sa*EAS possesses the ability to broadly re-enter *Streptomyces* spp. to antagonize competing species lacking immunity. However, our heterologous expression of *Sa*EAS in *S. venezuelae* and *S. coelicolor* likely rules out these two species as susceptible competitors because we observed no *Sa*EAS-dependent effects on their growth despite detecting abundant *Sa*EAS in the growth medium.

Our data also suggest that the *Sa*EAS gene is constitutively expressed by *Streptomyces*, which may have functional consequences during predator-prey interactions within the rhizosphere. For instance, EAS-secreting *Streptomyces* spp. may be able to rapidly synthesize alarmones within phagolysosomes or the cytoplasm upon phagocytosis by soil-dwelling predators such as the amoeba *Dictyostelium discoideum* (53). While luminal ATP in phagolysosomes does not have a clear role in bacterial killing (54), cytoplasmic alarmone synthesis may lead to energy depletion and serve as a defense mechanism to undermine predator fitness. Taken together, *Sa*EAS most likely functions as a sensor or effector to interact with other organisms in the environment, and its precise role warrants systematic investigation in future studies.

### The pro-survival alarmone (p)ppGpp is bactericidal when overproduced by toxic EAS proteins

An unexpected result that emerged from this study is that cytoplasmic expression of *Vp*EAS and *Aa*EAS, enzymes that exclusively produce (p)ppGpp, prevents colony formation in *E. coli* through transcriptional reprogramming and metabolic slowdown (1, 55, 56). Our *in vitro* biochemical assays and metabolite profiling experiments demonstrate that both enzymes specifically act on guanylate nucleotides and are therefore unlikely to act on other cellular targets. Although some toxSAS enzymes modify tRNA 3′ ends, these enzymes are generally unable to act on nucleotides. Conversely, *Vp*EAS and *Aa*EAS display robust (p)ppGpp synthetase activity, and to our knowledge, there is no essential RNA in *E. coli* with a 3′-terminal guanylate that would be suitable as a substrate for these enzymes.

Our previous work on Apk1, a (p)ppApp-specific toxSAS, offers insights into how alarmone overproduction causes a loss in bacterial viability (6). Apk1 exerts toxicity by depleting ADP/ATP pools as it rapidly synthesizes (p)ppApp, thereby disrupting numerous essential metabolic processes that rely on these nucleotides. Expression of the small alarmone hydrolases *Pa*SAH or Mesh1 partially rescues cells from Apk1-induced lethality, likely by converting a fraction of the (p)ppApp back into ADP/ATP. Notably, these hydrolases have *k*_cat_ values below 1,000 min^−1^ (7, 28), which is orders of magnitude slower than the 180,000 min^−1^ *k*_cat_ of Apk1, suggesting that ADP/ATP depletion, not alarmone accumulation, causes the decrease in viability.

Unlike (p)ppApp, (p)ppGpp is an endogenous signal that inhibits bulk transcription, which is the main consumption pathway for ribonucleotides (57). The concomitant inhibition of ribonucleotide biosynthesis by (p)ppGpp, including all pathways that feed into GTP production, prevents excessive ribonucleotide accumulation, thus permitting the biosynthesis of amino acids such as histidine and tryptophan under starvation (30, 58). Inhibition of GTP production by (p)ppGpp synthesis, which itself consumes GTP, drives GTP levels down to enable persister development in *B. subtilis* (59). Despite this established physiological role, this regulatory circuit poses a vulnerability in that a highly active (p)ppGpp synthetase would likely lead to total depletion of GTP. During amino acid starvation in *E. coli*, the long RSH enzyme RelA becomes activated by uncharged tRNA and converts GDP/GTP to (p)ppGpp, a process allosterically enhanced by pppGpp (23, 60). As GTP levels fall and pppGpp synthesis slows down, the phosphatase GppA converts pppGpp to ppGpp, reducing RelA activation and thus protecting the GTP pool from total depletion. Additionally, endogenous (p)ppGpp synthetases like *E. coli* RelA, *B. subtilis* Rel, and SasB have relatively low *k*_cat_ values (<1,000 min^−1^ when fully activated), and their activities are counterbalanced by (p)ppGpp hydrolases (23, 24, 61). We propose that *Vp*EAS and *Aa*EAS, like Apk1 and *Sa*EAS, have *k*_cat_ values far exceeding those of signaling enzymes and are not subject to regulatory feedback. Such overwhelming synthetase activity leads to rapid depletion of GTP, a nucleotide essential for transcription, translation, and ribonucleotide biosynthesis. The loss of bacterial viability is likely due to a lethal imbalance of cellular homeostasis caused by GTP depletion. Alternatively, *Vp*EAS and *Aa*EAS may be extremely stable in the *E. coli* cytoplasm, maintaining high ppGpp in otherwise viable cells, which could lead to a viable but non-culturable state. While we cannot formally distinguish between these two models, our data strongly suggest that the viability of alarmone-producing bacteria is defined by the synthetase’s catalytic efficiency and regulatory context, not the identity of the alarmone.

### Concluding remarks and limitations of the study

This study focuses on the systematic identification and biochemical characterization of EASs, including their enzymatic activities, substrate specificities, and secretion and processing via the Sec pathway. The enzymatic properties of *Sa*EAS suggest that it is adapted for low-substrate environments such as extracellular space. Overexpression in *E. coli* suggests that *Sa*EAS, *Vp*EAS, and *Aa*EAS could function as potential interbacterial toxins if they are internalized by other bacteria. Furthermore, loss of *E. coli* viability driven by (p)ppGpp synthesis challenges conventional views of (p)ppGpp as a strictly pro-survival signal. Overall, our work provides a foundation for future studies on the biology, evolution, and ecological functions of exported alarmone synthetases.

Beyond the scope of this initial study are the physiological roles of these enzymes. For example, we have not systematically studied the fitness consequences of deleting various EAS genes in their native organisms. We also did not identify any species capable of internalizing an EAS. Additionally, the physiological conditions under which *Sa*EAS produces extracellular alarmones and how these alarmones may be sensed by neighboring bacteria (e.g., by internalization or via surface receptors) also await further study.

## MATERIALS AND METHODS

### Bacterial strains, plasmids, and growth conditions

A full list of strains and plasmids used in this study can be found in Tables S2 and S3. *Escherichia coli* strains SM10 and ET12567 (pUZ8002::*bla*) were used for conjugation, strains BL21 CodonPlus(DE3)-RIL and B834(DE3) were used for protein expression, and strains K-12 MG1655 and BL21(DE3) were used for cell viability experiments. Strain DH5α λpir was used for maintenance of pBAD33mob and pJL-1, and all other plasmids were maintained in strain XL-1 Blue. *E. coli* strains were grown in liquid lysogeny broth (LB, containing 10 g L^−1^ NaCl, 5 g L^−1^ yeast extract, 10 g L^−1^ tryptone) at 37°C with shaking or on solid LB containing 1.5% (wt/vol) agar. *Streptomyces* strains were used for *Sa*EAS characterization. *Streptomyces albidoflavus* SM254, *Streptomyces coelicolor* M1146, and *Streptomyces venezuelae* ATCC 10712 were all grown in liquid tryptic soy broth (TSB; BD) at 30°C with shaking, on solid TSB containing 1.5% (wt/vol) agar or on solid mannitol-soya flour (MS; 20 g L^−1^ mannitol, 20 g L^−1^ soya flour) medium containing 2% (wt/vol) agar with or without 20 mM MgCl_2_ (used in interspecies conjugation). *Bacillus subtilis* strain PY79 was used for co-culture experiments with *S. albidoflavus*. This strain was grown in liquid LB medium at 30°C with shaking. *Vibrio* strains were used for *Vp*EAS characterization. *Vibrio parahaemolyticus* RIMD 2210633 was grown in liquid Marine-LB (MLB; LB containing 30 g L^−1^ NaCl) at 30°C with shaking or on solid MLB containing 1.5% (wt/vol) agar. *Vibrio cholerae* El Tor N16961 was grown in liquid LB at 37°C with shaking or on solid LB or M9 medium containing 1.5% (wt/vol) agar. For plasmid selection, media were supplemented with 50 µg mL^−1^ apramycin, 50 µg mL^−1^ kanamycin, 150 µg mL^−1^ carbenicillin, 20 µg mL^−1^ chloramphenicol, 50 or 100 µg mL^−1^ hygromycin B (100 µg mL^−1^ for *E. coli* ET12567, 50 µg mL^−1^ for *Streptomyces*), 15 µg mL^−1^ gentamicin, 50 µg mL^−1^ kanamycin, or 200 µg mL^−1^ trimethoprim. For protein expression, the medium was supplemented with 0.2% (wt/vol) L-arabinose, 250 µM–1 mM isopropyl β-D-1-thiogalactopyranoside (IPTG), 0.1% (wt/vol) L-rhamnose, or 40 µg mL^−1^ X-gal.

### DNA manipulation and plasmid construction

Primers were received from Integrated DNA Technologies. Molecular cloning reagents (restriction enzymes, Phusion polymerase, and T4 DNA ligase) were obtained from New England Biolabs. Sanger sequencing was performed by Genewiz Incorporated. All the vectors described in Table S3 were generated using standard restriction cloning procedures. Mutations were amplified through splicing by overlap extension PCR.

### Genetic manipulation and protein expression in *Streptomyces*

Deletion of *SaEAS* in *S. albidoflavus* SM254 was accomplished using the pCRISPomyces-2 plasmid containing an *SaEAS*-specific protospacer and a ∆*SaEAS* editing template, as described previously (20). Briefly, the protospacer (5′- CGGTCGACGCCTACGTGGAG −3′) was introduced into pCRISPomyces-2 using Golden Gate assembly. An in-frame *SaEAS* deletion template was generated through PCR amplification of ~1 kb upstream and downstream of the *SaEAS* gene. The flanks were then spliced using overlap extension PCR, and the resulting construct was ligated into *XbaI*-digested pCRISPomyces-2 containing the *SaEAS*-specific protospacer. The plasmid was transformed into *E. coli* ET12567 (pUZ8002::*bla*) by heat shock and subsequently introduced into *S. albidoflavus* using conjugal transfer, as described previously (62). Transconjugants were selected using 50 µg mL^-1^ apramycin and 50 µg mL^-1^ trimethoprim. The *SaEAS* deletion was verified by PCR and Sanger sequencing.

For protein expression in *Streptomyces* spp., either wild-type or mutant *SaEAS* or *gusA* (from *E. coli*) was amplified by standard PCR and cloned into the pIJ10257 vector under the control of the ermE* promoter or the native *SaEAS* promoter. The vector was then transformed into *E. coli* ET12567 (pUZ8002::*bla*) and subsequently conjugated into various *Streptomyces* species, as indicated.

### Protein expression and purification

pETDuet-1 or pET29b plasmids expressing wild-type or mutants of Apk1, Apk2, *Sa*EAS, or *Vp*EAS (both *V. parahaemolyticus* or *V. cholerae* homologs) were transformed into *E. coli* BL21 (DE3) CodonPlus (see Tables S2 and S3). For plasmids expressing Apk2, *Sa*EAS, or *Vp*EAS, liquid growth was minimized prior to inducing protein expression due to the accumulation of suppressors due to all three proteins being toxic to *E. coli*. A stock was made by resuspending BL21 transformants from solid media into liquid LB containing 15% (vol/vol) glycerol. A lawn of these cell lines was then plated and grown for 14–20 h overnight. Cells from the lawn were then resuspended into fresh liquid LB and standardized to a density of 1.5–2 OD_600_ mL^1^, creating a concentrated stock. For Apk1-expressing plasmids, a liquid overnight culture was used, as described previously (6). Both the liquid culture (Apk1) and concentrated stocks (Apk2-4) were then diluted 50-fold into fresh LB supplemented with antibiotics (~0.02–0.05 OD_600_). All cultures were then grown at 37°C with shaking. At mid-log phase (OD_600_ ~0.5), cultures expressing wild-type or mutant Apk1 were supplemented with 1 mM IPTG and kept at 37°C with shaking for an additional 4–5 h. For cultures expressing Apk2, *Sa*EAS, or *Vp*EAS, the culture was cooled to 18°C just before mid-log phase (OD_600_ ~0.4) and subsequently treated with 1 mM IPTG once the culture OD_600_ was above 0.5. Cells grown at 18°C were incubated for another 12–18 h post-induction. Cultures were then centrifuged, and cell pellets were resuspended in lysis buffer (50 mM Tris-HCl, pH 8.0, 300 mM NaCl, 10 mM imidazole). The resuspended cells were lysed by sonication (amplitude 35%, 30 second pulses for 2 min per sample) and subsequently centrifuged to collect the soluble cell lysate. Cleared lysates were applied to a Ni^2+^-nitrilotriacetic acid (Ni-NTA)-conjugated agarose gravity column. For Apk2, *Sa*EAS, or *Vp*EAS proteins, the column was washed with three column volumes (CVs) of lysis buffer. For experiments requiring free Apk1, an additional step involving denaturation and refolding was used to separate Apk1 from its cognate immunity protein, Tis1. Rather than lysis buffer, two CVs of denaturation buffer (20 mM Tris-HCl, pH 8.0, 300 mM NaCl, 10 mM imidazole, 8 M urea) were applied to the column. Next, the column was washed with “renaturing” buffer (20 mM Tris-HCl, pH 8.0, 300 mM NaCl) to achieve on-column refolding. For all proteins, column-bound His_6_-tagged proteins were eluted with lysis buffer containing 400 mM imidazole. All proteins were subjected to size-exclusion chromatography using a Superdex 75 10/300 GL or HiLoad 16/600 Superdex 200 pg column run with buffer containing 20 mM Tris-HCl, pH 8.0, 150 mM NaCl.

SasB and Apk2 were expressed and purified similarly to *Sa*EAS^ΔSP^ (L83M, D121A), as previously described. Apk1 was expressed and purified in complex with its cognate immunity protein, Tis1, also similarly using Ni-NTA resin. The His_6_-Apk1 bound to Ni-NTA resin was denatured with 8 M urea to enable removal of Tis1 and Apk1, then refolded by washing with urea-free buffer, as previously described (6, 63). All proteins were subjected to size-exclusion chromatography using a Superdex 75 10/300 GL or HiLoad 16/600 Superdex 200 pg column run with buffer containing 20 mM Tris-HCl, pH 8.0, 150 mM NaCl.

*Sa*EAS^ΔSP,D121A^-FLAG was expressed as an N-terminal His_6_-SUMO-tagged protein using *E. coli* BL21(DE3) harboring corresponding expression plasmids. Expression strains were grown in LB at 37°C to OD_600_ = 0.6 and induced with 200 µM IPTG for 2 h. Cells from a 50 mL expression culture were harvested by centrifugation at 4,000 *g* for 10 min and resuspended in 800 µL of TBS buffer (20 mM Tris-HCl, pH 8.0, 150 mM NaCl) containing Pierce Protease and Phosphatase Inhibitor. Cells were lysed by a Branson tip sonicator. The cleared lysate (20,000 × *g*, 10 min) was applied to 500 µL Ni-NTA resin equilibrated with the lysis buffer. The Ni-NTA resin was washed with 5 mL of TBS containing 25 mM imidazole and eluted with 2 mL TBS containing 300 mM imidazole. The eluate was concentrated using Amicon Ultra filters (10 kDa MWCO) and diluted with TBS in several iterations to remove imidazole. The protein was then treated with 0.1 mg Ulp1 at room temperature for 12 h. The mixture was subjected to reverse Ni-NTA, and the tag-free protein was recovered in the flow-through fraction. *Sa*EAS^ΔSP, D121A^-FLAG was further refined on a C5 reverse-phase HPLC (2.1 × 100 mm, 3 µm) with solvent A (0.1% trifluoroacetic acid, TFA) and a linear solvent B (0.1% TFA in 90% [vol/vol] acetonitrile in water) gradient, increasing from 30% to 70% over 8 min at 0.5 mL/min.

### Preparation of synthetase-containing cell lysates and *in vitro* activity assays

The BL21(DE3) strain harboring a pET29b expression plasmid for *Sa*EAS or *Vp*EAS and pSCrhaB2-CV expression plasmid for Apk2 or *Aa*EAS^CTD^ was grown at 37°C in 50 mL LB medium and induced with 0.2% (wt/vol) rhamnose at an OD_600_ of 0.3–0.4. Cells were harvested 2 h post-induction by centrifugation. Media was completely decanted, and cells were resuspended in 300 µL TBS buffer (20 mM Tris-HCl, pH 8.0, 150 mM NaCl) containing Pierce Protease and Phosphatase Inhibitor. The suspension was sonicated by a Branson tip at 12% amplitude for 3 × 10 seconds with sufficient cooling between pulses. The lysate was cleared at 20,000 × *g* for 10 min, and supernatant was used immediately for synthetase reactions.

Each synthetase reaction contained 50 mM HEPES-Na 7.5, 150 mM NaCl, 10 mM MgCl_2_, and 0.1 mM MnCl_2_. Final substrate concentrations were 5 mM for ATPγS and 1 mM for GTPγS (if included). A total of 5 µL of lysate was used in a 20 µL reaction. Each reaction was incubated at 37°C in a water bath for 5, 5, 60, and 60 min for lysates containing *Sa*EAS, *Vp*EAS, Apk2, or *Aa*EAS^CTD^, respectively. Reactions were also assembled containing 1 nM Apk1 or 50 µM SasB to specifically produce pppApp-2S and pppGpp-2S as peak-position standards. Reactions were stopped by taking 5 µL aliquots and diluting them in 200 µL of 0.1% cold acetic acid (CH_3_COOH), and the samples were resolved on a Capto HiRes Q 5/50 anion-exchange column (Cytiva). The column was run with buffer A (5 mM Tris-HCl, pH 8.0) and a linear buffer B (5 mM Tris-HCl, pH 8.0, 1 M NaCl) gradient, increasing from 10 to 50% over 10 mL.

### Immunoblotting and quantification of *Sa*EAS^ΔSP^-FLAG

*Streptomyces* cells expressing either wild-type or mutant *Sa*EAS were grown in 3 mL liquid TSB with 5 mM MgCl_2_ for 24 h (initial culture density: ~0.05 OD_600_ mL^-1^, diluted from ~30 OD_600_ mL^-1^ spore stock). The culture was centrifuged (15,000 × *g*) to separate the mycelium from the *Sa*EAS-containing spent medium. The mycelium was resuspended with 2× SDS-PAGE loading buffer and heated at 95°C for 5 min before being resolved on SDS-PAGE.

*Sa*EAS^ΔSP^-FLAG in the spent medium was TCA-precipitated to enable better detection. To each 400 µL spent medium sample, 100 µL 100% (wt/vol) TCA was added, and the mixed sample was incubated on ice for 10 min. The precipitate was pelleted at 4°C, 20,000 × *g,* and washed twice, each time by sonicating in 200 µL acetone and spinning at 20,000 × *g*. After removal of the second wash, the precipitate was dried on a 50°C heat block and dissolved in 20 µL of 2× SDS-PAGE loading dye. A total of 10 µL of this sample was resolved on 12% SDS-PAGE for immunoblotting. Proteins on SDS-PAGE were transferred to a PVDF membrane at 25 V for 10 min using the Bio-Rad Trans-Blot Turbo. The membrane was blocked overnight using Li-COR Intercept blocking buffer and incubated for 1 h with a polyclonal anti-FLAG antibody (Sigma F7425, 1:5,000 dilution) at room temperature on an orbital shaker, then washed twice for 5 min with TBST (20 mM Tris-HCl, pH 7.5, 150 mM NaCl, and 0.1% Tween-20) and incubated for 1 h with anti-rabbit horseradish peroxidase-conjugated secondary antibodies (Sigma, 1:5,000 dilution). After three additional washes in TBST, chemiluminescent substrate (Clarity Max, Bio-Rad) was used to develop the blot. Imaging was completed with the ChemiDoc Imaging System (Bio-Rad).

To quantify *Sa*EAS^ΔSP^-FLAG (Fig. S2B and C), the overexpression spent medium was diluted 1-to-5 or 1-to-10 in the spent medium from a culture of the ∆*SaEAS* strain that does not contain interfering anti-FLAG signal (See Fig. 3). The recombinant-protein standard, *Sa*EAS^ΔSP,D121A^-FLAG, was dissolved from lyophilized powder with 8 M urea and quantified based on absorbance at 280 nm. This stock solution was diluted in ∆*SaEAS* spent medium to 5.1, 1.5, and 0.51 nM final concentrations. All above-mentioned samples were subjected to TCA precipitation, SDS-PAGE, and anti-FLAG immunoblotting in duplicate. The immunoblot procedure was identical to the above-mentioned except the IRDye 800CW Anti-Rabbit IgG Goat Antibody (Li-COR 926-32211, 1:5,000 dilution) was used as secondary and the membrane was imaged and bands were quantified by densitometry using a Li-COR Odyssey imaging system.

### Measurement of pppApp-synthetase activity using colorimetric enzyme coupling

This experiment allows rapid quantification of pppApp-synthetase activity of spent medium and other protein preparations. One hundred microliter reactions were assembled on 96-well plates, each containing 50 mM HEPES-Na 7.5, 150 mM NaCl, 10 mM MgCl_2_, 0.1 mM MnCl_2_, 5 mM ATP, 3.75 mM phosphoenolpyruvate (PEP), 0.5 mM NADH, 20 U/mL myokinase (adenylate kinase), 20 U/mL pyruvate kinase (PK), and 20 U/mL lactate dehydrogenase. These reagents use AMP generated from pppApp synthesis to consume NADH, leading to a decrease in absorbance at 340 nm (*A*_340_) (see Fig. 3B). When applicable, reactions were assembled omitting myokinase to reveal background, ADP-producing activities, such as phosphatases or ATPases, also leading to NADH consumption. All reagents were prepared as concentrated stocks and diluted in an appropriate volume of water for 1.05× final concentration. After pre-incubation at 30°C for 5 min, 95 µL aliquots of this mixture were transferred, using a multi-channel pipette, to wells each containing 5 µL sample of unknown synthetase activity. The reactions were immediately incubated at 30°C in a Synergy H1 microplate reader (BioTek) with constant shaking, and *A*_340_ was measured every 1 min. Initial velocity in *A*_340_/min was generated for each reaction by linear regression. After correction for the corresponding “-myokinase” background, velocity in mM/min was calculated using an extinction coefficient of 3.88 per millimolar pppApp.

### Michaelis-Menten studies of *Sa*EAS^ΔSP^-FLAG

ATP, the only PPi donor used by *Sa*EAS^ΔSP^, is also used as a PPi acceptor for the synthesis of pppApp. Thus, the synthesis of any other alarmone occurs competitively with the concomitant synthesis of pppApp. To address this complication, we assumed that the *K_m_* of PPi-donor ATP is constant, independent of the identity of PPi-acceptor substrate. To distinguish the synthesis kinetics for all alarmones, we used anion-exchange chromatography to analyze all synthetase reactions, which contained 50 mM HEPES-Na 7.5, 150 mM NaCl, 5 mM free MgCl_2_, and 0.1 mM of MnCl_2_. AMP, ADP, GMP, and GDP were added as sodium salts, while ATP and GTP were added as 1:1 complex with Mg^2+^. Spent medium containing *Sa*EAS^ΔSP^-FLAG was diluted 1:10 in TBS containing 0.1 mg/mL bovine serum albumin as a 5× stock. Reactions were stopped after incubation at 37°C for 30 seconds, 2 min, or 5 min by taking 5 µL aliquots and diluting in 200 µL of 0.1% cold acetic acid.

Preliminary data (Fig. 3D) show that when ATP and guanylate nucleotides are present at comparable concentrations, *Sa*EAS^ΔSP^ prefers to synthesize (p)ppGpp. Using [GTP] = 5 mM and varying [ATP] at lower concentrations from 0.03 to 1 mM, ppGpp is the only detectable product. We used these conditions to determine the *K*_*m*_ for ATP as the PPi donor (Fig. 4D). To eliminate interference from GDP and ADP impurities in GTP and ATP reagents, these reactions contained an additional 20 U/mL PK and 2 mM PEP. Initial velocity in 30 seconds revealed that *K*_*m*, donor_ = 82 μM and *V*_max(pppGpp)_ = 110 µM/min. We next varied [ATP] from 0.5 to 10 mM in the absence of other nucleotide for pppApp synthesis (Fig. 4E). Assuming a constant *K*_*m*, donor_, ATP should saturate the donor site in these reactions. Thus, the Michaelis-Menten relationship provides the *K*_*m*_ for ATP as a PPi acceptor, *K*_*m*, aceptor(ATP)_ = 1.4 mM. Additionally, assuming saturation of both donor and acceptor sites, *V*_max(pppApp)_ = 114 µM/min. Using a different batch of spent-medium sample, from which the *Sa*EAS^ΔSP^-FLAG concentration was quantified, we determined pppApp-synthesis rate to be 23 ± 3 µM/min with 5 mM ATP by 0.30 nM *Sa*EAS^ΔSP^-FLAG. After factoring in the *K_m_* for ATP, this was equivalent to *K*_cat (pppApp)_ = 1.0 × 10^5^/min. *K*_cat_ for the synthesis of other alarmones shown in Fig. 4F was calculated to commensurate with their respective *V*_max_.

We next considered the competition among more than one PPi acceptors. The phosphorylation velocity of substrate *x* is:


(1) $$V_{x}=\frac{V_{\max(x)}\frac{\left[x\right]}{K_{m,\text{acceptor}(x)}}}{1+\sum_{i=a}^{n}\frac{\left[i\right]}{K_{m,\text{acceptor}(i)}}}\times \frac{\frac{\left[\text{ATP}\right]}{K_{m,\text{donor}}}}{1+\frac{\left[\text{ATP}\right]}{K_{m,\text{donor}}}}$$

in which *a* through *n* represents all substrates involved in competition. By comparing the production rate of pGpp, pApp, and ppApp relative to pppApp, we calculated the *V*_max_/*K*_*m*_ for GMP, AMP, and ADP (Fig. 4C and G). By comparing the production rate of pppGpp relative to pGpp, we calculated the *V*_max_/*K*_*m*_ for GTP (Fig. 4C).

We next used *V*_max(pppGpp)_ to calculate *K*_*m*, acceptor(GTP)_. To deconvolute *V*_max_ from *K*_*m*_ for GMP, AMP, and ADP, we calculated the fractional saturation (θ) of PPi-acceptor sites for ATP and *apo* acceptor sites using equations 2 and 3:


(2) $$\theta_{\text{acceptor(ATP)}}=\frac{V_{\text{pppApp}}}{V_{\max(\text{pppApp})}}\times \frac{1+\frac{\left[\text{ATP}\right]}{K_{m,\text{donor}}}}{\frac{\left[\text{ATP}\right]}{K_{m,\text{donor}}}}$$

(3) $$\theta_{\text{acceptor(apo)}}=\theta_{\text{acceptor(ATP)}}\frac{K_{m,acceptor(ATP)}}{\left[\text{ATP}\right]}$$

For reactions containing 5 mM ATP and 5 mM ADP, 5 mM AMP, or 0.5 mM GMP, we then calculated the acceptor-site occupancy and *K_m_* for the non-ATP substrate, *x*:


(4) $$\theta_{\text{acceptor}(x)}=1-\theta_{\text{acceptor(apo)}}-\theta_{\text{acceptor(ATP)}}$$

(5) $$K_{m,\;acceptor\text{(}x)}=\frac{\theta_{\text{acceptor(apo)}}}{\theta_{\text{acceptor}(x)}}[x]$$

*V*_max_ and *K*_cat_ for the synthesis of ppApp, pApp, or pGpp were hence calculated based on their respective *V*_max_/*K*_*m*_. Quantification of ppGpp is challenging because *Sa*SAS produces pppApp concomitantly with ppGpp, and anion-exchange chromatography cannot separate these products (see Fig. 4C). We therefore decided not to report *K*_*m*, acceptor(GDP)_.

### Reporter assay for *Vibrio* and *Streptomyces*

For Fig. S2A: S. *albidoflavus* strains harboring pIJ10257 expressing *gusA* (from *E. coli*) under the control of either the constitutively active ermE* promoter or the native *SaEAS* promoter were grown on solid MS medium supplemented with 50 µg mL^-1^ hygromycin B and 40 µg mL^-1^ X-gal.

### Crystallization and structure determination

The crystal structure corresponds to a *Sa*EAS_G36-P248_ L83M, D121A double mutant (*Sa*EAS*^∆SP^). The L83M and D121A mutations were generated for selenomethionine incorporation and to minimize toxicity during protein overexpression, respectively. This double mutant was expressed with some modifications to the protocol described above for other SAS proteins. The vector used to express the *Sa*EAS mutant was transformed into the methionine auxotroph *E. coli* B834 (DE3). As described for Apk2, *Sa*EAS, or *Vp*EAS proteins, multiple cycles of growth in liquid media were avoided to limit the accumulation of suppressor mutations. We made stocks of the B834 cells directly by resuspending transformants from solid media into LB containing glycerol. Instead of a liquid stationary-phase culture to be used for back-dilution, a lawn from the stock was grown on solid media at 37°C. Cells from the lawn were then resuspended directly into sterile liquid SelenoMet Medium (Molecular Dimensions) containing L-selenomethionine in place of L-methionine to a culture density of ~2–3 OD_600_ mL^-1^. To limit contamination by methionine in the LB media, the resuspended culture was centrifuged and washed with fresh SelenoMet media twice, creating a concentrated stock, similar to an overnight stationary-phase culture. The concentrated stock was diluted 50-fold into 1–2 L fresh L-selenomethionine-containing SelenoMet Medium, and the cells were grown at 37°C with shaking. The remainder of the protein expression is the same as described above and took place at 18°C. Following gel filtration, the selenomethionine-incorporated *Sa*EAS mutant was concentrated to 15 mg/mL by spin (5 kDa MWCO, MilliporeSigma). Hanging drop vapor diffusion was used to test crystallization at 23°C using the MCSG1-4 (Anatrace) sparse matrix screens. The crystals used for structure determination grew in 0.1 M imidazole-HCl, pH 8, 1 M sodium citrate, and the crystals were frozen using ethylene glycol as cryoprotectant. Diffraction data were collected at 100 K on the ADSC Q315r detector at 19-BM beamline of the Structural Biology Center at the Advanced Photon Source. The data were processed using HKL3000 suite. The processed intensity data (I and σI) were converted to amplitudes (F and σF), and 5% of the data were randomly selected for omission from downstream analyses using TRUNCATE before the structure was solved using MOLREP with an AlphaFold2 model of *Sa*EAS (64, 65). The structure was initially refined using refmac5.5 and manually adjusted using Coot (66, 67). TRUNCATE, MOLREP, and refmac5.5, all used for solving the structure, are parts of the HKL3000 suite. The iterative refinement process was carried out using Phenix.refine with TLS parameterization for B factors and Coot until the structure converged to *R*_work_/*R*_free_ values of 0.238/0.276 with reasonable stereochemistry validated by MolProbity, PROCHECK, and PDB validation (68–70). Throughout the refinement, the same selected 5% data were removed and used for *R*_free_ calculations, which were used for monitoring the progress of the refinement. The final structure was deposited with the PDB ID of 8VX3 (71). Please see Table S1 for details. Structural figures were generated using ChimeraX (72).

### Metabolite extraction from *E. coli* for nucleotide profiling on anion-exchange columns

Stationary-phase cultures of *E. coli* cells harboring the synthetase-expressing pSChraB2-CV plasmids were diluted into 50 mL of LB for an initial density of 0.02 OD_600_. Protein expression was induced with 0.2% rhamnose at OD_600_ = 0.3–0.4. Ten milliliters of culture was retrieved, from which cells were collected onto 0.45 µm hydrophilic PVDF membrane by vacuum filtration. Cells were immediately washed with 1 mL 160 mM NaCl and submerged into a tube containing ice-cold lysis solvent composed of methanol, acetonitrile, and water (40:40:20, vol/vol/vol). The tube was sonicated to ensure suspension of cells before the membrane was removed. This suspension, corresponding to 1.0 (OD_600_ × mL) cells, was diluted to 4 mL in 10 mM Tris-HCl, pH 8.0, and subsequently centrifuged at 20,000 × *g* to remove insoluble matter. The supernatant was subjected to a 0.22 µM filter, degassed, and applied to a Capto HiRes Q5/50 anion-exchange column (Cytiva). The column was run with buffer A (5 mM Tris-HCl, pH 8.0) and a linear buffer B (5 mM Tris-HCl, pH 8.0, 1M NaCl) gradient, increasing from 10 to 50% over 12 mL, to elute negatively charged metabolites.

### Growth curves, toxicity assays, and bacterial competition experiments

To assay for growth inhibition by expression of SYNTH-domain proteins (Fig. 1; Fig. S1 and S5), stationary-phase cultures of cells harboring pSCrhaB2-CV expression plasmid for the indicated proteins were diluted in 10-fold serial dilutions to a concentration of 10^-6^. Ten microliters of undiluted cells and each dilution (10^0^–10^-6^) were then spotted onto an LB agar plate containing 200 µg mL^-1^ trimethoprim and 0.1% rhamnose (wt/vol). For *V. cholerae* spot plates, cultures containing empty pBAD33mob or pBAD33mob expressing wild-type or mutant *Vc*RelV cultures were similarly diluted and plated on LB agar containing 20 µg mL^-1^ chloramphenicol and 0.2% (vol/vol) L-arabinose.

For the combined growth curve and cell viability experiment, *E. coli* cells harboring the synthetase-expressing pSChraB2-CV plasmids were diluted from overnight stationary-phase starter, cultured, and induced as described above for metabolite-extraction experiments. The culture was sampled at appropriate time points for OD_600_ measurement. Cells were pelleted at 20,000 × *g* and resuspended in fresh LB. This suspension was serially diluted to 10^−6^ in fresh LB, and 10 µL of each dilution was plated onto LB agar containing 200 µg mL^−1^ trimethoprim for CFU enumeration. The CFU were enumerated after 18 h of growth on solid media at 37°C.

For the *Bacillus* and *Streptomyces* competition experiment, cultures of *B. subtilis* or *S. albidoflavus* were grown in liquid LB or TSB at 30°C with shaking for 24 h, respectively. The cultures were centrifuged, and the cell pellets were washed with fresh TSB twice. The cultures were then mixed in a 1:1 ratio, and 10 µL of the mixture was spotted onto a TSB agar plate. Ten microliters (10 µL) of *Streptomyces* cells alone was also directly plated onto a TSB agar plate. The samples were grown for 3 days and imaged daily.

### Bioinformatics analyses

#### Phylogenetic distribution of Apk1-like and SaEAS-like sequences

For Apk1-like sequences, we used jackhmmer to conduct an iterative search procedure of the profile HMM of the Apk1 toxin domain (residues 251–460) of the UniProtKB database for related sequences in bacteria (31, 73). After two iterations, we identified 2,959 sequences (File S2). Sequences with >95% similarity were removed using CD-HIT, yielding 1,369 sequences for downstream analysis (Files S3 and S4) (74). For *Sa*EAS-like sequences, we used phmmer to search the UniProtKB database for *Streptomyces* sequences with homology to full-length *Sa*EAS (31). The search yielded 152 sequences that were used for downstream analysis. For both data sets, we aligned all sequences using MAFFT (gap open penalty: 2; offset value: 0.123) and generated a Jukes-Cantor neighbor-joining tree (no outgroup). Clusters were identified as either (i) ≥20 sequences with ≥40% sequence identity—six total clusters identified (A–F) (Apk1 homologs tree)—or (ii) ≥5 sequences with ≥35% sequence identity—five total clusters (*Sa*EAS homologs tree) identified. iTOL was used for tree visualization (75). Sequences from each cluster were analyzed for the presence of signal sequences, transmembrane domains, and protein homology using SignalP 6.0, Phobius, HHpred, and HMMER (39, 41, 76).

## Data Availability

All data supporting the findings of this study are available within the article and its associated supplemental information. X-ray crystallographic coordinates and structure factor files are available from the PDB: SaEAS_G36-P248_ D121A, L83M double mutant (8VX3). Files containing all Apk1- and SaEAS-like sequences can be found in the supplemental information.

## SUPPLEMENTAL MATERIAL

File S1 mbio.02227-25-s0001.txt List of SaEAS-like sequences in *Streptomyces* spp.

File S2 mbio.02227-25-s0002.txt List of 2,959 Apk1/Tas1 homologs identified using jackhmmer search.

File S3a mbio.02227-25-s0003.txt List of all non-redundant gene containing Apk1/Tas1 homologous sequences used for phylogenetic analysis.

File S3b mbio.02227-25-s0004.txt List of final 1,369 non-redundant Apk1/Tas1 homologous sequences used for phylogenetic analysis in Fig. 6.

File S4 mbio.02227-25-s0005.txt List of full-length Apk1/Tas1 homologs from File S3b.

Supplemental figures and tables mbio.02227-25-s0006.docx Figures S1-S5, Tables S1-S3, and captions for Files S1 to S4.

ASM does not own the copyrights to Supplemental Material that may be linked to, or accessed through, an article. The authors have granted ASM a non-exclusive, world-wide license to publish the Supplemental Material files. Please contact the corresponding author directly for reuse.
