# Supplementary material for: Bacteria export alarmone synthetases that produce (p)ppApp and (p)ppGpp
Source: mBio. 2025 Nov 12;16(12):e02227-25. doi: 10.1128/mbio.02227-25 (PMC12691689; doi:10.1128/mbio.02227-25)
Supplement: Supplemental figures and tables — Figures S1-S5, Tables S1-S3, and captions for Files S1 to S4. [file mbio.02227-25-s0006.docx]

**Supplemental Material**

**Bacteria export alarmone synthetases that produce (p)ppApp and (p)ppGpp**

Shehryar Ahmad^1,2,3,8^, Andrea G. Guedez^4,8^, Berti Manisa^4^, Abdulmalik Adewale^4^, Kara K. Tsang^5^, Vanessa Schiefer^2,3^, Nathan P. Bullen^2,3^, Harmy Thakar^2,3^, Youngchang Kim^6^, Boyuan Wang^4*^, and John C. Whitney^2,3,7*^

^1^Temerty Faculty of Medicine, University of Toronto, Toronto, Ontario M5S1A8, Canada

^2^Michael DeGroote Institute for Infectious Disease Research, McMaster University, Hamilton, Ontario L8S 4K1, Canada

^3^Department of Biochemistry and Biomedical Sciences, McMaster University, Hamilton, Ontario L8S 4K1, Canada

^4^Department of Pharmacology, UT Southwestern Medical Center, Dallas, Texas 75235, USA

^5^Department of Infection Biology, London School of Hygiene and Tropical Medicine, London WC1E 7HT, United Kingdom

^6^Structural Biology Center, X-ray Science, Argonne National Laboratory, Lemont, IL 60439, USA

^7^David Braley Centre for Antibiotic Discovery, McMaster University, Hamilton, ON L8S 4K1, Canada

^8^Equal Contribution

**Supplementary Figure S1. Cytoplasmic expression of a signal peptide-deficient variants of *Vc*RelV and *Vp*EAS is toxic to bacteria.**

**Supplementary Figure S2. Expression and quantification of recombinant *Sa*EAS^DSP^-FLAG.**

**Supplementary Figure S3. Production of extracellular *Sa*EAS by *S. albidoflavus* does not affect the growth of *B. subtilis*.**

**Supplementary Figure S4. *Sa*EAS** **homologs are widespread in *Streptomyces* species and are fused to diverse protein export domains.**

**Supplementary Figure S5. *Aa*EAS is a type VII secretion system (T7SS)-associated (p)ppGpp synthetase that is bactericidal in *E*. *coli*.**

**Supplementary Table S1. X-ray data collection and refinement statistics.**

**Supplementary Table S2. Strains used in this study.**

**Supplementary Table S3. Plasmids used in this study.**

**Supplementary File 1. List of *Sa*EAS-like sequences in *Streptomyces* spp (.txt). Associated with Figure S4A.**

**Supplementary File 2. List of all 2,959 Apk1/Tas1 homologs hits identified through *jackhmmer* search (.txt).**

**Supplementary File 3. List of all clusters with < 95% similarity and curated 1,369 Apk1/Tas1 homologous sequences used for phylogenetic analysis. Two files within a .zip folder. Clusters are listed as “Supplementary_File_3a” and final sequences are listed as “Supplementary_File_3b”. Associated with Figure 6A.**

**Supplementary File 4. List of full-length Apk1/Tas1 homologs from Supplementary File 3b.**

**Supplemental references**


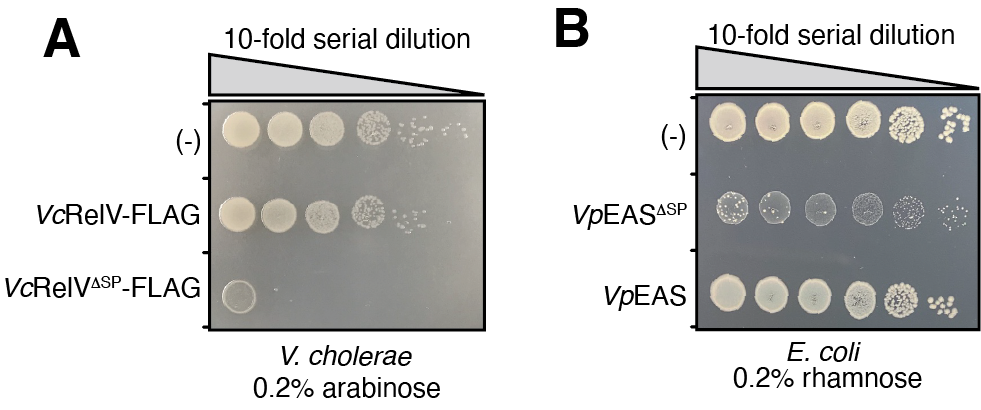


**Figure S1. Cytoplasmic expression of a signal peptide-deficient variants of *Vc*RelV and *Vp*EAS is toxic to bacteria.** Overnight growth of either *V*. *cholerae* (**A)** or *E. coli* (**B**) expressing the indicated synthetase on inducer-containing media.


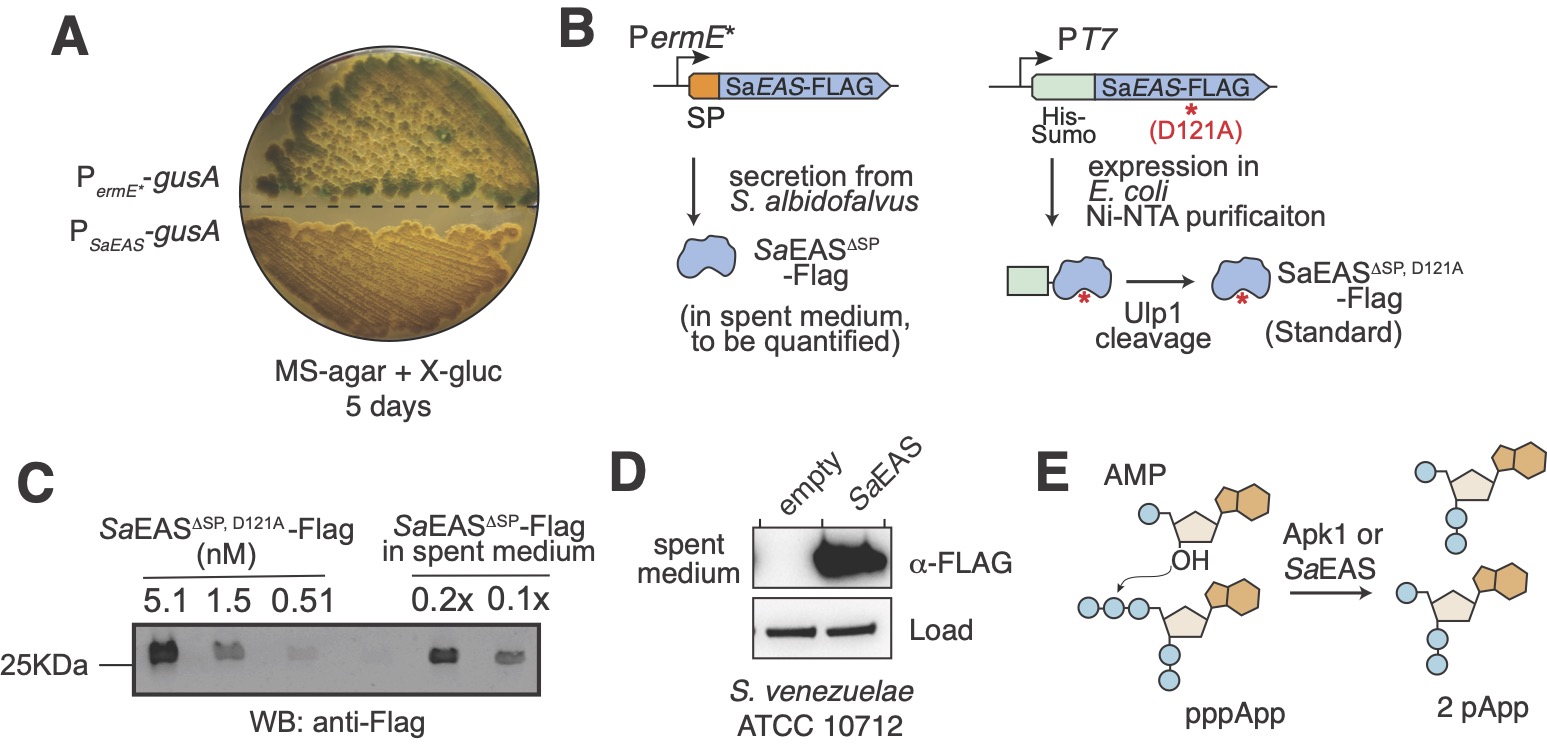


**Figure S2. Expression and quantification of recombinant *Sa*EAS^DSP^-FLAG.** (**A**) Growth of *S*. *albidoflavus* strains expressing *gusA* (*E*. *coli* glucuronidase) under the control of a constitutively active *ermE** promoter (P*_ermE_*_*_) or the native *SaEAS* promoter (P*_SaEAS_*). Strains were struck on MS agar and grown for 120 hours. (**B**) Expression systems used for *Sa*EAS. The *Sa*EAS^DSP^-FLAG construct is expressed under a constitutively active promoter and secreted into the *S. albidoflavus* supernatant (left). The *Sa*EAS^DSP,D121A^-FLAG active-site mutant is overexpressed as an N-terminal His-Sumo fusion under the control of a T7 promoter in *E. coli* (right). The His-Sumo tag was proteolytically removed to provide a standard for *Sa*EAS^DSP^-FLAG quantification. (**C**) Immunoblot-quantification of *Sa*EAS^DSP^-FLAG using the D121A mutant as an internal standard. (**D**) Anti-FLAG immunoblot of spent medium sample from a *S. venezuelae* culture expressing *Sa*EAS-FLAG. (**E**) Diagram showing the pyrophosphokinase activity of *Sa*EAS or Apk1 using pppApp as the PPi donor and AMP as the acceptor, giving rise to 2 pApp.


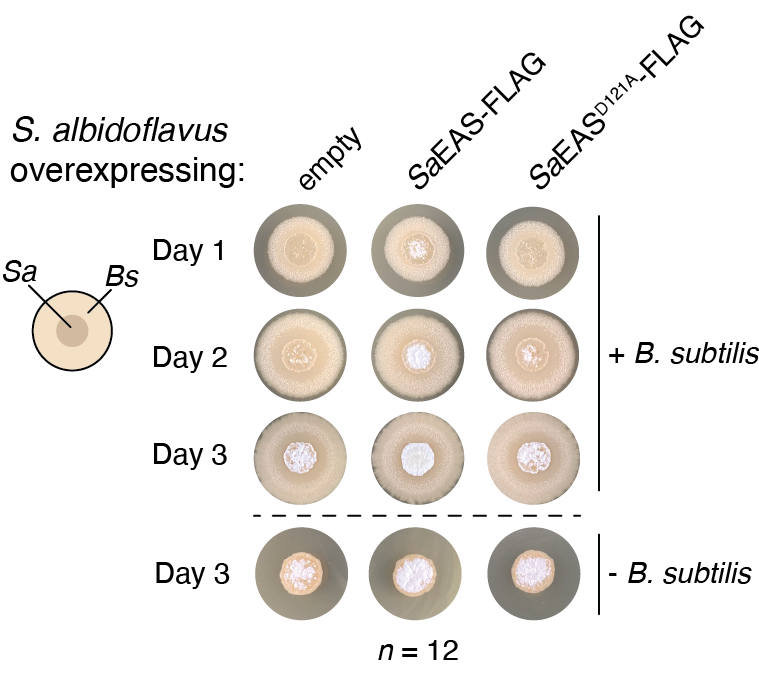


**Figure S3. Production of extracellular *Sa*EAS by *S. albidoflavus* does not affect the growth of *B. subtilis*.** *S. albidoflavus* (*Sa*) overexpressing the indicated proteins was spotted on TSB agar either in co-culture with *B*. *subtilis* (*Bs*) (top) or alone (bottom). *Sa* grows in the centre of the spot, whereas *Bs* grows in an outer ring. Images were taken after 3 days of incubation. The data are representative of *n* = 12 biological replicates across twelve individual experiments.


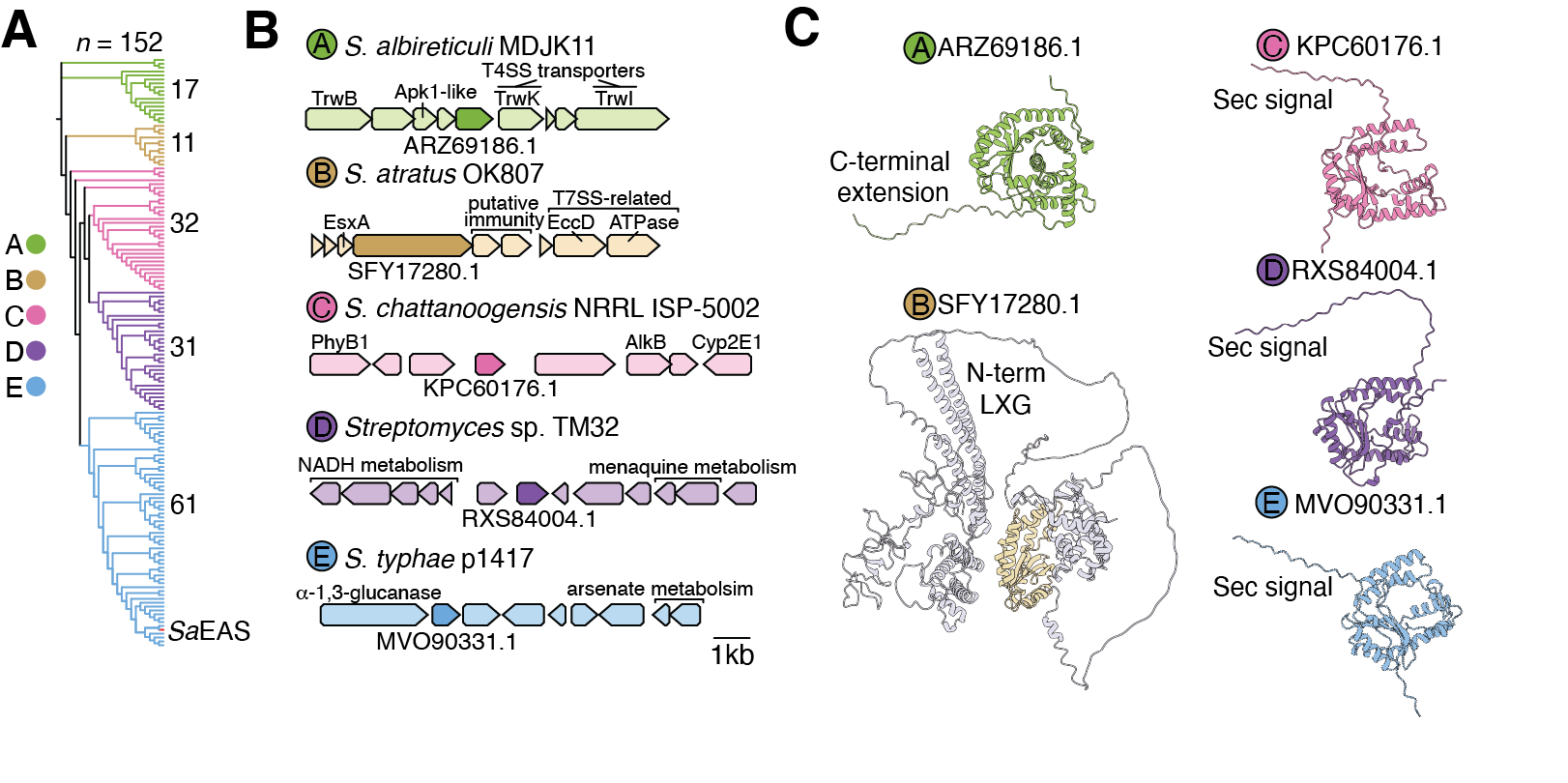


**Figure S4. *Sa*EAS** **homologs are widespread in *Streptomyces* species and are fused to diverse protein export domains.** (**A**) Phylogenetic distribution of 152 *Sa*EAS homologous sequences in *Streptomyces*. Five clusters (A-E) with their respective sizes are shown. Clusters are defined as sequences with ≥35% similarity. Position of *Sa*EAS sequence is highlighted in red. (**B**) Genomic neighborhoods of five *Sa*EAS homologs from each cluster. *SaEAS*-like genes are coloured in a darker colour shade and are labeled with their protein ID. Annotations are based on protein homology predictions (1). Full list of sequences is available in Supplementary File 1. (**C**) Alphafold3-predicted models of representative *Sa*EAS homologs from (**B**). The synthetase domain is coloured in all structures with N- or C-terminal extensions labeled.


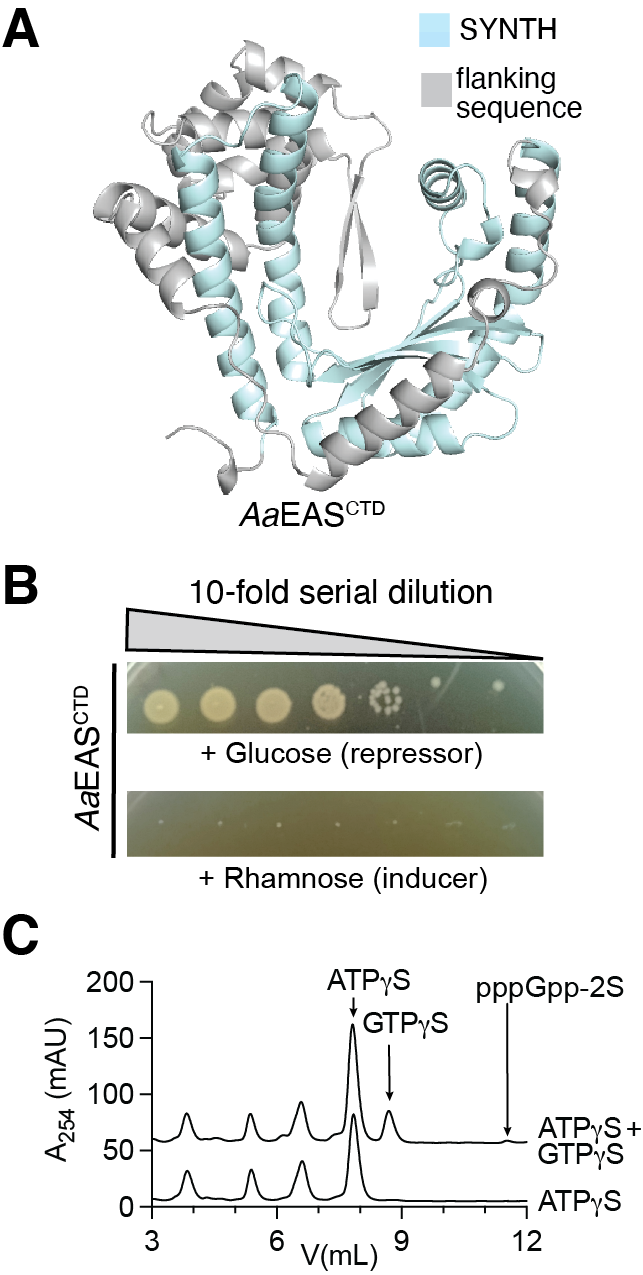


**Figure S5. *Aa*EAS is a type VII secretion system (T7SS)-associated (p)ppGpp synthetase that is bactericidal in *E*. *coli*.** (**A**) Alphafold3-predicted model of the C-terminal domain (CTD) of *Aa*EAS (*Aa*EAS^CTD^). The structural elements in gray highlight an extension of the SYNTH domain (blue). The CTD consists of the extension and SYNTH domain (as shown in Figure 6C). (**B**) Overnight growth of *E. coli* harboring *Aa*EAS^CTD^ expression vector on inducer- or repressor-containing LB agar. (**C**) Anion-exchange traces of 5mM ATPγS alone or together with 1 mM GTPγS upon 1-hr treatment with cell lysates from *E*. *coli* expressing *Aa*EAS^CTD^.

**Table S1. X-ray data collection and refinement statistics.**

|  | ***Sa*EAS** |
| --- | --- |
| **Data Collection** |  |
| Space group | P2_1_ |
| Cell dimensions |  |
| *a, b, c* (Å) | 74.98, 132.62, 68.44 |
| *α*, *β*, *γ* (°) | 90.0, 89.77, 90.0 |
| Wavelength (Å) | 0.97933 |
| Resolution (Å) | 47.57 – 2.40 (2.44-2.40) ^a^ |
| No. of reflection | 53335 (2646) |
| *R*_merge_^b^ | 0.127 (1.29) |
| *I* / σ(*I*) | 12.86 (1.11) |
| Completeness (%) | 98.6 (97.1) |
| Redundancy | 4.4 (4.0) |
| CC_1/2_ | 0.990 (0.418) |
|  |  |
| **Refinement** |  |
| Resolution (Å) | 47.57 – 2.40 (2.44-2.40) |
| No. reflections used | 51099 (2458) |
| R_Free_ Data used (%) | 5.22 |
| *R*_work_ / *R*_free_ (%)^c^ | 23.8/27.6 |
| Twining  Operator/fraction | *-h,-k,l* /0.470 |
| No. protein chains/ASU | 4 |
| No. atoms |  |
| Protein | 6186 |
| Ligand/ion | 23 |
| Water | 106 |
| *B*-factors (Å^2^) | 78.3 |
| Protein | 78.5 |
| Ligand/ion | 64.7 |
| Water | 48.5 |
| r.m.s deviations |  |
| Bond lengths (Å) | 0.002 |
| Bond angles (°) | 0.406 |
| Molprobity statistics^d^ |  |
| Clashscore | 4.78 |
| Rotamer outlier | 7.62 |
| Ramachandran plot |  |
| Favored/outlier (%) | 96.95/0.0 |
| PDBID | 8VX3 |

^a^Values in parentheses correspond to the highest resolution shell.

^b^*R*_merge_ = Σ Σ |*I*(*k*) - <*I*>|/ Σ *I*(*k*) where *I*(*k*) and <*I*> represent the diffraction intensity values of the individual measurements and the corresponding mean values. The summation is over all unique measurements.

^c^*R*_work_ = Σ ||*F*_obs_| - *k*|*F*_calc_||/|*F*_obs_| where *F*_obs_ and *F*_calc_ are the observed and calculated structure factors, respectively. *R*_free_ is the sum extended over a subset of reflections excluded from all stages of the refinement.

^d^As calculated using MOLPROBITY (2).

**Table S2. Strains used in this study.**

| Organism | Genotype | Description | Reference |
| --- | --- | --- | --- |
| *S*. *albidoflavus* SM254 | Wild-type |  | Gift from Christine Salomon |
|  | pIJ10257::ECK1612 (Hyg^R^) | Wild-type strain with pIJ10257 expressing *E*. *coli* *gusA* under the control of the *ermE** promoter inserted at a neutral chromosomal site | This study |
|  | pIJ10257::P-Salbus254_1145-ECK1612 (Hyg^R^) | Wild-type strain with pIJ10257 expressing *E*. *coli* *gusA* under the control of the *SaEAS* promoter inserted at a neutral chromosomal site | This study |
|  | ∆Salbus254_1145 | Strain harboring in-frame *SaEAS* deletion | This study |
|  | ∆Salbus254_1145  pIJ10257 (Hyg^R^) | ∆*SaEAS* strain with pIJ10257 inserted at a neutral chromosomal site | This study |
|  | ∆Salbus254_1145  pIJ10257::Salbus254_1145-FLAG (Hyg^R^) | ∆*SaEAS* with pIJ10257 expressing *SaEAS* (with a C-terminal FLAG epitope) inserted at a neutral chromosomal site | This study |
|  | ∆Salbus254_1145  pIJ10257::Salbus254_1145_E34-CT-FLAG (Hyg^R^) | ∆*SaEAS* with pIJ10257 expressing *SaEAS*_∆SS_ (with a C-terminal FLAG epitope) inserted at a neutral chromosomal site | This study |
|  | ∆Salbus254_1145  pIJ10257::Salbus254_1145_D121A-FLAG (Hyg^R^) | ∆*SaEAS* with pIJ10257 expressing *SaEAS** (attenuated mutant) (with a C-terminal FLAG epitope) inserted at a neutral chromosomal site | This study |
| *S*. *coelicolor* M1146 | Wild-type |  | Gift from Gerard Wright |
|  | pIJ10257 (Hyg^R^) | Wild-type strain with pIJ10257 inserted at a neutral chromosomal site | This study |
|  | pIJ10257::Salbus254_1145-FLAG (Hyg^R^) | Wild-type strain with pIJ10257 expressing *SaEAS* inserted at a neutral chromosomal site | This study |
| *S*. *venezuelae* ATCC 10712 | Wild-type |  | Gift from Gerard Wright |
|  | pIJ10257 (Hyg^R^) | Wild-type strain with pIJ10257 inserted at a neutral chromosomal site | This study |
|  | pIJ10257::Salbus254_1145-FLAG (Hyg^R^) | Wild-type strain with pIJ10257 expressing *SaEAS* (with a C-terminal FLAG epitope) inserted at a neutral chromosomal site | This study |
| *B*. *subtilis* PY79 | Wild-type |  |  |
| *V*. *parahaemolyticus* RIMD 2210633 | Wild-type |  | Gift from Dor Salomon |
| *V*. *cholerae* El Tor N16961 | Wild-type |  | Gift from Tobias Dörr |
| *E*. *coli* XL-1 Blue | *recA1* *endA1* *gyrA96 thi-1 hsdR17 supE44 relA1 lac* [F´ *proAB lacI*^q^ Z∆*M15* Tn*10* (Tet^R^)] | Plasmid maintenance strain |  |
| *E. coli* DH5α λpir | *endA1 hsdR17 glnV44 (= supE44) thi-1 recA1 gyrA96 relA1 φ80dlacΔ(lacZ)M15 Δ(lacZYA-argF)U169 zdg-232::Tn10 uidA::pir+* | Plasmid maintenance strain | Novagen |
| *E. coli* SM10 λpir | *thi thr leu tonA lac Y supE recA::RP4-2-Tc::Mu* |  |  |
| *E*. *coli* BL21 CodonPlus (DE3) -RIL | F^-^ *ompT* *hsdS*_B_(r_B_^-^ m_B_^-^)  dcm^+^ Tet^r^ *gal* λ(DE3) *endA* Hte [*argU* *ileY* *leuW* Cam^r^] | Protein expression strain | Novagen |
| *E*. *coli* B834 (DE3) | F^-^*ompT* *hsdS*_B_(r_B_^-^ m_B_^-^) *gal* *dcm* *met* (DE3) | Methionine auxotroph used for protein expression | Novagen |
| *E*. *coli* K-12 MG1655 | F^-^ lambda^-^ *ilvG*^-^ *rfb*-50 *rph*-1 | Expression strain for toxicity assays |  |
| *E*. *coli* ET12567 | *F- dam-13::Tn9 dcm-6 hsdM hsdR zjj-202::Tn10 recF143 galK2 galT22 ara-14 lacY1 xyl-5 leuB6 thi-1 tonA31 rpsL136 hisG4 tsx-78 mtl-1 gln* pUZ8002::*bla* Cam^R^ Amp^R^ | Required for plasmid conjugation into *Streptomyces* spp. | Gift from Gerard Wright |

**Table S3. Plasmids used in this study.**

| Plasmid | Relevant features | Reference |
| --- | --- | --- |
| pSCrhaB2-CV | Expression vector with *rhaB* promoter, Tmp^R^ | (3) |
| pPSV39-CV | Expression vector with *lacI*, *lacUV5* promoter, C-terminal VSV-G tag, Gm^R^ | (4) |
| pETDuet-1 | Co-expression vector with *lacI*, T7 promoter, N-terminal His_6_ tag in MCS-1, Amp^R^ | Novagen |
| pET29b | Expression vector with *lacI*, T7 promoter, C- terminal His_6_ tag, Kan^R^ | Novagen |
| pIJ10257 | *Streptomyces* integration vector for constitutive expression of genes under control of *ermE** promoter2 ΦBT1 phage integration site, contains *oriT* from RK2, Hyg^R^ | (5) |
| pCRISPomyces-2 | CRISPR/Cas9 vector for chromosomal mutagenesis in *Streptomyces* species, Apr^R^ | (6) |
| pBAD33mob | Expression vector in *E*. *coli* and *Vibrio* species, Cam^R^ | (7, 8) |
| pJL-1 | Native *lacZ* (VC2338)-integrating vector in *Vibrio* species, Amp^R^ | (7, 9) |
| pSCrhaB2-CV::PA14_01140_M251-CT | Expression vector for *tas1*_251-460_ | (10) |
| pSCrhaB2-CV::BACCAC_01148 (A1212C)_D339-CT-VSV-G | Expression vector for C-terminal VSV-G-tagged *apk2*_339-538_ | This study |
| pSCrhaB2-CV::BACCAC_01148 (A1212C)_D339-CT_ E365A-VSV-G | Expression vector for C-terminal VSV-G-tagged *apk2*_339-538_ E365A point mutant | This study |
| pSCrhaB2-CV:: Salbus254_1145_E34-CT-VSV-G | Expression vector for C-terminal VSV-G tagged *SaEAS*_34-251_ | This study |
| pSCrhaB2-CV:: Salbus254_1145_E34-CT-FLAG | Expression vector for C-terminal FLAG tagged *SaEAS*_34-251_ | This study |
| pSCrhaB2-CV:: Salbus254_1145_E34-CT_D121A -VSV-G | Expression vector for C-terminal VSV-G tagged *SaEAS*_34-251_ D121A point mutant | This study |
| pSCrhaB2-CV:: VP1295-VSV-G | Expression vector for C-terminal VSV-G tagged *VpEAS* | This study |
| pSCrhaB2-CV:: VP1295_A23-CT-VSV-G | Expression vector for C-terminal VSV-G tagged *VpEAS*_23-257_ | This study |
| pSCrhaB2-CV:: VP1295_A23-CT_E186A -VSV-G | Expression vector for C-terminal VSV-G tagged *VpEAS*_23-257_ E186A point mutant | This study |
| pETDuet-1:: His_6_-Salbus254_1145 ::BACCAC_01146-VSV-G | Expression vector for N-terminal His_6_-tagged *SaEAS* and C-terminal VSV-G tagged *aph1* | This study |
| pETDuet-1::His_6_-Salbus254_1145_E34-CT_D121A ::BACCAC_01146-VSV-G | Expression vector for N-terminal His_6_-tagged *SaEAS_34-251_* D121A point mutant and C-terminal VSV-G tagged *aph1* | This study |
| pETDuet-1:: His_6_-Salbus254_1145_E34-CT_D121A_L83M-FLAG ::BACCAC_01146-VSV-G | Expression vector for N-terminal His_6_-tagged *SaEAS_34-251_* L83M, D121A double mutant and C-terminal VSV-G tagged *aph1* | This study |
| pETDuet-1:: His_6_-VP1295_A23-CT:: BACCAC_01146-VSV-G | Expression vector for N-terminal His_6_-tagged *VpEAS_23-257_* and C-terminal VSV-G tagged *aph1* | This study |
| pETDuet-1:: His_6_-VP1295_A23-CT_E186A:: BACCAC_01146-VSV-G | Expression vector for N-terminal His_6_-tagged *VpEAS_23-257_* E186A point mutant and C-terminal VSV-G tagged *aph1* | This study |
| pETDuet-1::His_6_-PA14_01140_M251-CT::PA14_01130 | Expression vector for N-terminal His_6_-tagged *apk1_251-460_* and *tis1* | (10) |
| pET29b::Salbus254_1145-His_6_ | Expression vector for C-terminal His_6_-tagged *SaEAS* | This study |
| pET29b::VP1295-His_6_ | Expression vector for C-terminal His_6_-tagged *VpEAS* | This study |
| pET29b:: VP1295_A23-CT_E186A-His_6_ | Expression vector for C-terminal His_6_-tagged *VpEAS_23-257_* E186A point mutant | This study |
| pIJ10257:: Salbus254_1145-FLAG | Expression vector for C-terminal FLAG-tagged *SaEAS* for use in *Streptomyces* spp. | This study |
| pIJ10257:: Salbus254_1145_D121A-FLAG | Expression vector for C-terminal FLAG-tagged *SaEAS* D121A point mutant for use in *Streptomyces* spp. | This study |
| pIJ10257:: Salbus254_1145_E34-CT-FLAG | Expression vector for C-terminal FLAG-tagged *SaEAS_34-251_* for use in *Streptomyces* spp. | This study |
| pIJ10257::P*_ermE_*_*_-ECK1612 | Expression vector for *E*. *coli* *gusA* under the control of a constitutively active *ermE** promoter | This study |
| pIJ10257::P_Salbus254_1145_- ECK1612 | Expression vector for *E*. *coli* *gusA* under the control of the *SaEAS* gene promoter | This study |
| pCRISPomyces-2::Salbus254_1145_protospacer::∆Salbus254_1145_HR | *SaEAS* deletion allele and *SaEAS*-specific protospacer in pCRISPomyces-2 | This study |
| pBad33mob:: VC1224-FLAG | Expression vector for C-terminal FLAG-tagged *VcEAS* (*V*. *cholera* homolog) | This study |
| pBad33mob:: VC1224_V24-CT-FLAG | Expression vector for C-terminal FLAG-tagged *VcEAS_23-259_* (*V*. *cholera* homolog) | This study |
| pSAS035 | Rhamnose-inducible expression vector for *B*. *subtilis* SasB | This study |
| pSAS065 | Rhamnose-inducible expression vector for *Aa*EAS^CTD^ | This study |
| pR3-1 | Constitutive expression vector for human Mesh1 with promoter and terminator sequences from *E. coli lpp*; | This study |
| pR3-1(E65A) | Constitutive expression vector for human Mesh1^E65A^ with promoter and terminator sequences from *E. coli lpp*; | This study |

**Supplemental references**

1. Soding J, Biegert A, Lupas AN. 2005. The HHpred interactive server for protein homology detection and structure prediction. Nucleic Acids Res 33:W244-8.

2. Chen VB, Arendall WB, 3rd, Headd JJ, Keedy DA, Immormino RM, Kapral GJ, Murray LW, Richardson JS, Richardson DC. 2010. MolProbity: all-atom structure validation for macromolecular crystallography. Acta Crystallogr D Biol Crystallogr 66:12-21.

3. Cardona ST, Valvano MA. 2005. An expression vector containing a rhamnose-inducible promoter provides tightly regulated gene expression in Burkholderia cenocepacia. Plasmid 54:219-28.

4. Rietsch A, Vallet-Gely I, Dove SL, Mekalanos JJ. 2005. ExsE, a secreted regulator of type III secretion genes in Pseudomonas aeruginosa. Proc Natl Acad Sci U S A 102:8006-11.

5. Hong HJ, Hutchings MI, Hill LM, Buttner MJ. 2005. The role of the novel Fem protein VanK in vancomycin resistance in Streptomyces coelicolor. J Biol Chem 280:13055-61.

6. Cobb RE, Wang Y, Zhao H. 2015. High-efficiency multiplex genome editing of Streptomyces species using an engineered CRISPR/Cas system. ACS Synth Biol 4:723-8.

7. Murphy SG, Alvarez L, Adams MC, Liu S, Chappie JS, Cava F, Dorr T. 2019. Endopeptidase Regulation as a Novel Function of the Zur-Dependent Zinc Starvation Response. mBio 10.

8. Gu D, Liu H, Yang Z, Zhang Y, Wang Q. 2016. Chromatin Immunoprecipitation Sequencing Technology Reveals Global Regulatory Roles of Low-Cell-Density Quorum-Sensing Regulator AphA in the Pathogen Vibrio alginolyticus. J Bacteriol 198:2985-2999.

9. Butterton JR, Beattie DT, Gardel CL, Carroll PA, Hyman T, Killeen KP, Mekalanos JJ, Calderwood SB. 1995. Heterologous antigen expression in Vibrio cholerae vector strains. Infect Immun 63:2689-96.

10. Ahmad S, Wang B, Walker MD, Tran HR, Stogios PJ, Savchenko A, Grant RA, McArthur AG, Laub MT, Whitney JC. 2019. An interbacterial toxin inhibits target cell growth by synthesizing (p)ppApp. Nature doi:10.1038/s41586-019-1735-9.
